# Improved Radiofrequency Safety Modelling in MRI Using In Vivo Measurements of Brain Conductivity

**DOI:** 10.1002/nbm.70335

**Published:** 2026-06-23

**Authors:** Guillaume Paillart, Zhongzheng He, Grecia Romero, Pauline Ferry, Khalid Ambarki, Paul Soullié, Jacques Felblinger, Freddy Odille

**Affiliations:** ^1^ IADI (U1254) Université de Lorraine and Inserm Nancy France; ^2^ ICube (UMR 7357) University of Strasbourg and CNRS Strasbourg France; ^3^ Healtis Nancy France; ^4^ Siemens Healthcare SAS Courbevoie France; ^5^ CIC‐IT 1433, Inserm Université de Lorraine and CHRU Nancy Nancy France

**Keywords:** *B*_1_ mapping, electromagnetic simulation, MR electrical properties tomography (MR‐EPT), MRI safety, specific absorption rate

## Abstract

Tissue electrical properties are required by electromagnetic simulation software to conduct radiofrequency (RF) safety studies. Values are commonly taken from existing databases of tissue properties, where brain conductivity was measured ex vivo. We hypothesize that using in vivo brain conductivity values, as reported in the recent literature on in vivo MRI measurements, can improve the accuracy of such simulations. Sixteen subjects were scanned at 3T to obtain experimental maps of the transmit RF field, $B_{1}^{+}$, of the head. Electromagnetic simulations were performed using biomodels with varying morphologies, using both a conventional ($\sigma_{\mathrm{ex}\;\text{vivo}}=0.46\;S/m$) and a modified brain conductivity ($\sigma_{\text{in vivo}}=0.70\;S/m$). A framework was developed to process the simulated $B_{1}^{+}$ fields, including a systematic method to combine the two excitation ports of the transmit coil model (accounting for different load impedances between simulation and experiment), geometric alignment, and scaling of the simulated fields, allowing a quantitative comparison of complex $B_{1}^{+}$ maps of the brain with experimental maps. Specific absorption rate (SAR) maps were also estimated by different methods, including a novel $B_{1}^{+}$‐derived formula. Normalized root‐mean‐squared errors between simulation and experiment, in the brain, were approximately 6% for $B_{1}^{+}$ magnitude. While head geometry mainly impacted the accuracy of $B_{1}^{+}$ magnitude, with errors up to 11% (*p* = 0.007) between the best fitting human model and the worst one, brain electrical conductivity mainly impacted $B_{1}^{+}$ phase, with errors reduced by 48% when using $\sigma_{\text{in vivo}}$ instead of $\sigma_{\mathrm{ex}\;\text{vivo}}$ (*p* = 0.0004). The agreement in average brain SAR, between simulation and experiment, was also improved, with differences reduced from 62% ($\sigma_{\mathrm{ex}\;\text{vivo}}$) to 22% ($\sigma_{\text{in vivo}}$). Using brain conductivity values from recent in vivo studies improves the accuracy of RF safety modelling.

**Trial Registration:**

ClinicalTrials.gov identifier: NCT04645628.

## Graphical abstract

A workflow for the comparison of simulated and measured $B_{1}^{+}$ maps was developed and applied to a study on human tissue (brain) electrical conductivity, investigating differences between conductivity values from ex vivo and in vivo measurements. The result showed a significantly better agreement when using in vivo conductivity values.

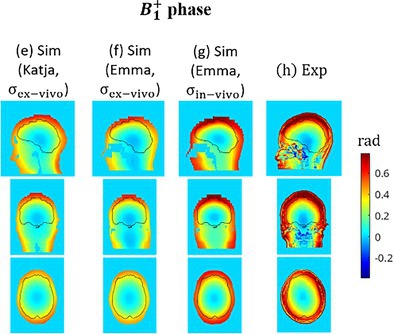

**Abbreviations**
AFI: actual flip angle imaging
ASTM: American Standards of Technical Material
CSF: cerebrospinal fluid
DICOM: digital imaging and communications in medicine
DREAM: dual refocusing echo acquisition mode
FLASH: fast low angle shot
GM: gray matter
GRAPPA: generalized autocalibrating partial parallel acquisition
ICNIRP: International Commission on Nonionizing Radiation Protection
MAPE: mean absolute percentage error
MRI: magnetic resonance imaging
NRMSE: normal root mean square error
RF: radiofrequency
SAR: specific absorption rate
UTE: ultrashort echo time
WM: white matter

## 1 Introduction

Clinical magnetic resonance imaging (MRI) requires exposing the patient to radiofrequency (RF) fields. Part of the transmitted electromagnetic energy is absorbed by the tissues and converted to thermal energy, causing a risk of excessive heating of tissues. The ICNIRP (International Commission on Nonionizing Radiation Protection) publishes guidelines [1, 2] for limiting the specific absorption rate (SAR), which is a quantitative measure of electromagnetic exposure. At 3T, whole‐body SAR can be estimated from the input and reflected power measured by the whole‐body transmit coil. Estimating local SAR during actual patient scans is challenging. Partial body weights can be used to estimate regional SAR in body parts such as the head, but local SAR cannot be monitored directly. A recent study showed that the local SAR limits defined in the safety standards are always reached before whole‐body SAR limits, both at 7T and at 3T [3]. Local SAR depends on the applied fields, but also on the patient‐specific morphology and tissue electrical properties, that is, conductivity and permittivity. Assessing accurate local SAR values is essential to ensure the safety of patients implanted with medical devices. RF induced local burns around implanted medical devices are described in case reports, especially for medical devices including leads [4, 5, 6, 7]. Also many studies have reported the significant heating rises when investigating the safety of medical devices during MRI exposure: hip joints [8], orthopedic implants [9], pacemaker leads [10], ECG leads [11], neurostimulation systems [12] among others.

Simulation is commonly used to estimate electromagnetic field maps in realistic scenarios, and ultimately to calculate SAR maps. This involves modeling both the field source, that is, the transmit coil (geometry, materials and electronic components), and the medium, including patient geometry, number of tissue classes, and tissue electrical properties. Simulation is not perfectly accurate, because (i) there are uncertainties in many model elements and (ii) a compromise should be made between model accuracy and computation time. A recent study showed that current simulation models are adequate for predicting total SAR in phantoms, but result in large underestimation of whole‐body SAR in human subjects, up to 84%, compared to that derived from reflected power measurements by the transmit coil [13].

In particular, the normal values of tissue electrical properties in vivo are not well known, neither is their variability in the population. Extensive measurements have been made with ex vivo human samples and animal studies [14], providing databases of tissue electrical properties, which are commonly used by the community. Emerging techniques have allowed in vivo measurements by MR electrical property mapping [15]; however, a large variability can be observed between studies. Differences are expected between ex vivo and in vivo values and have been demonstrated in animal studies [3]. Recently, in vivo conductivity in the brain was measured by MRI at 3T in 17 healthy volunteers, and was consistently higher than ex vivo values [16]. Similar results were also found in two volunteers at 7T [17]. In the former study at 3T, significant changes related to age, sex, or fat fraction were also found in various organs. A review [18] on tissue electrical properties measurement along different methods also pinpoints that, at a given frequency, some results are quite different from the reference values (e.g., at 109 MHz, gray matter conductivity of 0.68 S/m using a dielectric probe against 0.57 S/m from [19]). The study [20] questioned the brain electrical properties values from reference database [14]; however, the calculated values were too uncertain (standard deviations too high) to draw a significant conclusion. In the first challenge on MR electrical property tomography reconstruction, brain conductivity values from an in vivo dataset were also found to be higher than the known ex vivo values by most of the 35 participants [21]. Should these in vivo results be confirmed by other studies, it would be interesting to know how these changes impact SAR simulation results.

To quantify the accuracy of SAR simulation, several studies have compared simulation results to actual measurements. In phantoms, temperature measurements can serve as a reliable ground truth, provided that geometry, electrical and thermal properties are known, and electromagnetic simulation is complemented by a thermal solver. However, in patients, temperature is also influenced by the body thermal regulation system, so it cannot be directly used as a ground truth. Alternatively, transmit RF field maps, called $B_{1}^{+}$ maps, have been used for comparison [22]. At 3T, transmission is commonly achieved with a whole‐body two‐channel birdcage coil. $B_{1}^{+}$ magnitude maps, which can be directly measured by MRI, have been the primary focus of comparison in most studies [23, 24, 25]. $B_{1}^{+}$ phase can also be estimated under the transceive phase assumption [26]. However, for a direct comparison, several challenges need to be addressed, including (1) a combination of two excitation ports accounting for differences in load impedances between simulation and experiment and (2) proper scaling of the simulated $B_{1}^{+}$ field, accounting for unknown factors in the transmission chain. Finally, SAR maps can also be estimated from complex $B_{1}^{+}$ maps, under some assumptions [27].

The objective of this work was to assess the impact of electrical properties on RF safety modeling of the brain at 3T. To this end, the accuracy of $B_{1}^{+}$ simulation (and subsequent SAR estimates) was assessed both in a phantom and in subject experiments, with conventionally used (ex vivo) electrical properties, and with hypothesized higher in vivo conductivity values, as observed for the brain in a recent study [16]. The impact of model geometry was also investigated for comparison. Accuracy was assessed by the error between experimental and simulated $B_{1}^{+}$ maps (both magnitude and phase), and the accuracy of brain SAR values was subsequently estimated using various methods, including a novel $B_{1}^{+}$‐derived SAR estimation.

## 2 Methods

### 2.1 MR Imaging Protocol

The phantom consisted of a 166 × 140 × 70 mm^3^ volume filled in with a gelled‐saline solution with known properties, as described in the ASTM standard [28]. It was scanned using a 3T MAGNETOM Prisma scanner (Siemens Healthineers, Erlangen, Germany). RF transmission and reception were achieved with the system's whole‐body two‐port coil. Quadrature excitation was used as the two‐port transmission mode, as recommended in international standards for MRI safety assessment protocols. The experimental $B_{1}^{+}$ field was obtained using the vendor's |$B_{1}^{+}$| mapping sequence (presaturated 2D turbo‐FLASH sequence [29], TE/TR = 2 ms/30 s, 224 × 224 matrix, 29 axial slices, 1.2 × 1.2 × 7.5 mm^3^ resolution) and the phase of $B_{1}^{+}$ was estimated to be half the phase (i.e., transceive phase assumption [30]) of the research application UTE SpiralVIBE sequence (3D coronal volume, nonselective 3D excitation, stack‐of‐spirals sampling, TE/TR = 0.05/4.3 ms, sagittal orientation, 224 × 224 matrix, 160 slices, 1.2 × 1.2 × 1.2 mm^3^ resolution, flip angle 3°). Right before scanning, a conductometer (HANNA HI99301) measurement was performed in order to check that the gel had the desired electrical conductivity (0.47S/m at 128 MHz, also measured following the method mentioned in [31], section 2.2).

Sixteen healthy volunteers were scanned on the same MR scanner (seven women and nine men). This study was approved by an ethics committee, and informed written consent was obtained. The average age was 43±17 years old (range 23–73), height was 171±8cm, and weight was 72±11kg (body mass index: 25 ± 3 kg/m^2^). Characteristics for each volunteer are given in Table S1. Transmission was still with the whole‐body coil, but reception was achieved with a clinical 20‐channel head–neck coil. For each volunteer, the same two sequences as in the phantom were used for $B_{1}^{+}$ magnitude and phase mapping. Furthermore, a 3D T_1_‐weighted magnetization‐prepared rapid gradient echo sequence (axial orientation, 256 × 256 × 176, 1 × 1 × 1 mm^3^, TE/TR = 2.45/2300 ms, TI = 900 ms, GRAPPA factor 3) was acquired in order to perform brain segmentation using SPM software (SPM12, London, UK). Among the CSF, gray matter and white matter tissue classes, only the gray and white matter masks were used and grouped into one whole brain mask for subsequent analysis.

### 2.2 Electromagnetic Simulation

Numerical modeling was performed using CST Studio Suite 2022 software (Dassault Systèmes, Paris, France). A birdcage model was used, which was designed and previously tuned to the body coil of the 3T Prisma scanner [32]. It was designed based on the vendor's specifications of the coil geometry, including diameter/lengths, location of capacitors, and excitation ports. This model was a high pass birdcage model with 32 legs, with capacitors (32 for each two end‐rings) of 60pF, and the corresponding resonant frequency was 123.259 MHz. Two excitation ports were located 90° apart and were excited separately. The finite integration technique was used with a hexahedral mesh, with a shut‐off criterion defined as a −40dB accuracy defining that a steady‐state has been reached (according to the field energy monitor). The excitation ports were defined with an impedance normalization of 50 Ω. Electric (**E**) and magnetic (**H**) fields were generated (for each excitation port), as well as S‐parameters, with an input power normalized to 1 W. A hexahedral/cubic adaptive mesh was generated.

The phantom was modelled by a 166 × 140 × 70 mm^3^ cuboid. The simulation was run both with the known electrical properties of the ASTM F2182‐e2 gel (conductivity σ=0.47S/m, relative permittivity $\varepsilon_{r}=80$) and with an incorrect conductivity value (σ=0.24S/m), in order to quantify its impact on $B_{1}^{+}$ and SAR.

Three human models from the CST Family 2.0 (Katja, Emma, and Hugo) were used and their main properties are summarized in Table 1. Those three biomodels were chosen among several available models because they had different body mass indexes and geometries, providing a small‐sized, a medium‐sized, and a large‐sized adult biomodels. In some of these biomodels, only a global brain region was defined, that is, there were no distinct classes for white and gray matter, therefore, a single “average” brain conductivity was considered in all cases. For each biomodel, two simulations were run: the first one with brain conductivity set to $\sigma_{\mathrm{ex}\;\text{vivo}}=0.46\;S/m$ (default value in CST, based on commonly used values from the literature [14, 19, 33]), and the second one with brain conductivity set to $\sigma_{\text{in vivo}}=0.70\;S/m$ (adult average brain value measured in vivo from a previous study [16]). For each biomodel, the center of the head was placed at the isocenter of the birdcage model.

**TABLE 1:** Characteristics of each biomodel used for electromagnetic simulation in the study.

Model name	Sex	Age (years)	Size (cm)	Mass (kg)	Number of tissue classes	Resolution (mm^3^)
Katja	Female	43	163	62	71	1.775 × 1.775 × 1.21
Emma	Female	26	170	81	40	0.98 × 0.98 × 1
Hugo	Male	38	180	103	31	1 × 1 × 1

We performed an additional study to investigate the validity of averaging white matter (WM) and gray matter (GM) for $B_{1}^{+}$ and SAR maps. The Hugo model was used, as it was the only one from the CST family subset in which those two classes were defined separately. Two simulations were run, the first one using conductivities from the state‐of‐the‐art ex vivo database $\left(\sigma_{\mathrm{WM};\mathrm{ex}\;\text{vivo}}=0.34\;S/m,\sigma_{\mathrm{GM};\mathrm{ex}\;\text{vivo}}=0.58\;S/m\right)$, the second one using the conductivities from the in vivo measurements [16] $\left(\sigma_{\mathrm{WM};\text{in vivo}}=0.66\;S/m,\sigma_{\mathrm{GM};\text{in vivo}}=0.74\;S/m\right)$. As Hugo model includes an additional cerebellum tissue class (not included in Emma and Katja models), the ex vivo conductivity value was used in the first simulation $\left(\sigma_{\text{Cereb};\mathrm{ex}\;\text{vivo}}=0.82\;S/m\right)$. A study [34] based on five human cerebella measured a mean GM volume of 90.4 cm^3^ (72% of the total volume) and a mean WM volume of 33.1 cm^3^ (26% of the total volume), for a total mean volume of 125 cm^3^. As both tissue have a similar mean density [19], we applied an in vivo WM and GM weighted conductivity value for the cerebellum, resulting in $\sigma_{\text{Cereb};\text{in vivo}}=0.70\;S/m$. The agreement of $B_{1}^{+}$ and SAR maps from one of the subjects (subject 10) was compared when using the multiclass brain model or the single‐class brain model ($\sigma_{\mathrm{ex}\;\text{vivo}}=0.46\;S/m$ and $\sigma_{\text{in vivo}}=0.70\;S/m$). The relative permittivity values in the multiclass brain model were respectively 53.1 for white matter, 74.4 for gray matter and 81.1 for cerebellum.

### 2.3 Processing of the Simulated and Experimental $B_{1}^{+}$ Fields

**FIGURE 1:**
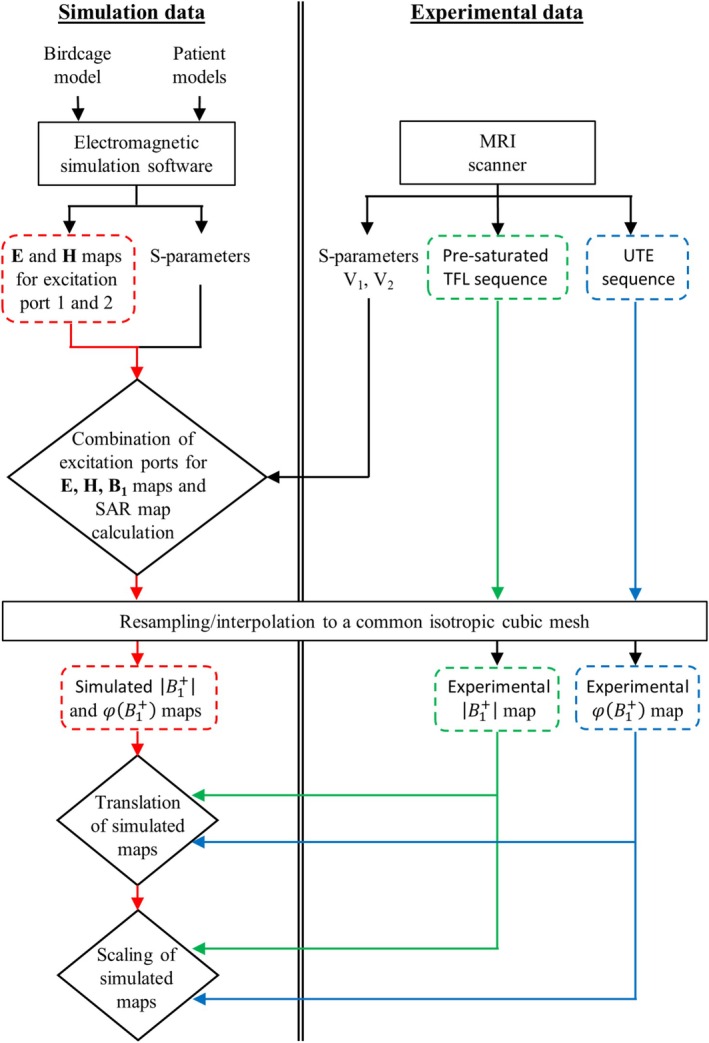
Workflow for obtaining both experimental and simulated $B_{1}^{+}$ maps, including combination of the two excitation ports, interpolation/resampling, alignment, and scaling.

To compare experimental and simulated $B_{1}^{+}$ maps, a processing workflow was implemented as described in Figure 1. This workflow was the same for the phantom and the volunteer data. Details are given hereafter.

#### 2.3.1 Combination of Excitation Ports Matching the Load Impedance

The simulation and the experimental setup may differ significantly because of differences in the load impedance. This may be due to the inaccuracies in the patient model or coil model, in particular some electrical and nonelectrical components present in the field exposure of the MRI scanner (gradient coils, magnet, and patient table) were not modelled. In a two‐port excitation setting, this should be taken into account with a corrected combination of excitation ports in the simulation. We define $V_{1,\mathrm{app}}^{\mathrm{sim}}$ and $V_{2,\mathrm{app}}^{\mathrm{sim}}$, the voltages applied to the excitation ports in the simulation, and $V_{1,\mathrm{app}}^{\exp}$ and $V_{2,\mathrm{app}}^{\exp}$, the voltages applied to the excitation ports in the experiment. Setting $V_{1,\mathrm{app}}^{\mathrm{sim}}$ to have a magnitude of 1 V and a zero phase, the corrected combination was calculated as:

(1) $$V_{2,\mathrm{app}}^{\mathrm{sim}}=\frac{V_{2,\mathrm{app}}^{\exp}}{V_{1,\mathrm{app}}^{\exp}}R\times V_{1,\mathrm{app}}^{\mathrm{sim}}$$

where *R* is a dimensionless factor calculated from the simulated and experimental S‐parameters. The rationale and detailed calculation of *R* is given in Appendix 1.

The total simulated $B_{1}^{+}$ field is therefore obtained by first calculating $B_{1}^{+}$ for each port, that is, $B_{1,\text{port}\;j}^{+}=\mu_{0}\left(H_{j,x}+i\;H_{j,y}\right)/2$, with $\mu_{0}$ the vacuum permeability; second combining the two partial $B_{1}^{+}$ fields, that is, $B_{1}^{+\mathrm{sim}}=\alpha_{1}B_{1,\text{port}1}^{+\mathrm{sim}}+\alpha_{2}.B_{1,\text{port}2}^{+}$, with $\alpha_{1}$ and $\alpha_{2}$ defined by

(2) $$\alpha_{n}=\frac{V_{n,\mathrm{app}}^{\mathrm{sim}}}{V_{1,\mathrm{app}}^{\mathrm{sim}}}$$

The total SAR map, with an averaging mass of 1 g, was also generated using the same port combination. All simulated maps were exported at isotropic resolution of 1 × 1 × 1 mm^3^.

#### 2.3.2 Interpolation and Translation Correction

Images coordinates from the 3D isotropic UTE sequence were extracted from DICOM information (ImagePositionPatient and ImageOrientationPatient tags) and were used to define a reference cubic mesh. All field maps ($B_{1}^{+}$ magnitude and phase from experiment and simulation, SAR maps) and brain masks were interpolated to the reference mesh (linear interpolation kernel).

In volunteer experiments, the center of the brain may not be exactly at the center of the birdcage coil. Therefore, for each volunteer, the simulated $B_{1}^{+}$ map was translated by $x_{c}^{\exp}-x_{c}^{\mathrm{sim}}$, with $x_{c}^{\exp}$ and $x_{c}^{\mathrm{sim}}$ the center of mass of the brain in the experiment and simulation, respectively.

#### 2.3.3 Scaling of Simulated $B_{1}^{+}$ Fields Maps

Experimental $B_{1}^{+}$ maps were expressed in μT units by dividing the flip angle map (in radian units), given by the $\left|B_{1}^{+}\right|$ mapping sequence, by γτ, with γ the proton gyromagnetic ratio and τ the effective duration of the RF pulse (alternatively, it can be scaled to the $\left|B_{1,\mathrm{rms}}^{+}\right|$ value of that sequence). An arbitrary reference value for the $B_{1}^{+}$ phase was chosen to be the phase at the center of the region of interest, that is, the phase at the center of the phantom or at the center of mass of the brain for volunteers (coordinate $x_{c}$), so $\varphi \left(B_{1}^{+}\left(x_{c}\right)\right)$ was subtracted from the raw $B_{1}^{+}$ phase.

Simulated $B_{1}^{+}$ maps were scaled to account for unknown gains in the transmission chain using the following (complex) scaling:

(3) $$B_{1}^{+\mathrm{sim},\;\text{scaled}}=B_{1}^{+\mathrm{sim}}\frac{B_{1}^{+\exp}\left(x_{c}\right)}{B_{1}^{+\mathrm{sim}}\left(x_{c}\right)}$$

The scaling factor was computed independently for each simulation run, that is, a different factor was used for each biomodel and for each conductivity value.

### 2.4 Voxel‐Wise Quantitative Comparison

In the phantom, the simulated, scaled $B_{1}^{+}$ map obtained with each conductivity value was compared quantitatively by computing the normalized root‐mean‐squared error (NRMSE) with respect to the experimental $B_{1}^{+}$ map, both in magnitude and phase. The experimental $B_{1}^{+}$ phase map (estimated from the UTE sequence) may be inaccurate at the edges of the field of view, where it is likely affected by $B_{0}$ inhomogeneities. Therefore, NRMSE calculations in the phantom were restricted to a mask based on the simulated phantom, smaller than the experimental phantom, and centered at the scanner isocenter.

For each volunteer, the simulated, scaled $B_{1}^{+}$ map obtained with each biomodel and each conductivity value was also compared quantitatively by computing NRMSE with respect to the experimental $B_{1}^{+}$ map (magnitude and phase). To have a fair comparison between the different biomodels, which have different head sizes, NRMSE was computed in a common brain mask, which was the intersection of the experimental brain mask (from the actual volunteer) and the brain masks from each of the three biomodels (Katja, Emma, and Hugo).

SAR maps were subsequently estimated. In volunteer data, SAR was estimated using the biomodel which gave the best match between simulated and experimental $B_{1}^{+}$ maps (i.e., lowest NRMSE in the brain). Table 2 gives the main electromagnetic properties for experimental SAR map calculation. Those properties were defined as spatially uniform over the whole region, either the phantom or the whole head. In the case of experimental SAR maps in subjects, a subject‐specific brain mask was applied and, for the simulated SAR maps, a biomodel‐specific brain mask. All the masks were eroded prior to applying Equation (4) in order to avoid boundaries errors due to the numerical differentiation kernel (erosion with a sphere of 7.2‐mm radius). In the case of the Hugo biomodel, which has several tissue classes for the brain, the brain mask is defined as the merging of white matter, gray matter, and cerebellum masks. The ground truth SAR map is unknown; however, SAR may be estimated from the experimental $B_{1}^{+}$ maps using the following formula (see derivation in Appendix 2):

(4) $${\mathrm{SAR}}_{B_{1}^{+}-\text{derived}}\approx \frac{2\sigma }{\rho_{m}{\left|\mu_{0}\kappa \right|}^{2}}\left({\left|\frac{\partial B_{1}^{+}}{\partial z}\right|}^{2}+{\left|\frac{\partial B_{1}^{+}}{\partial x}-i\frac{\partial B_{1}^{+}}{\partial y}\right|}^{2}\right)$$

with $\kappa =\sigma +\mathrm{i\omega}\varepsilon_{0}\varepsilon_{r}$ the complex admittivity, σ the local conductivity, $\varepsilon_{r}$ the local relative permittivity, $\varepsilon_{0}$ the vacuum permittivity, and $\rho_{m}$ the local mass density ($\rho_{m}=1000\;\mathrm{kg}/m^{3}$ in the phantom, $\rho_{m}=1045\;\mathrm{kg}/m^{3}$ in the brain). We computed the average SAR in the region of interest (above‐mentioned mask in the phantom, brain in the volunteers), using the corresponding properties. Spatial derivatives in Equation (4) were calculated using Savitzky–Golay filtering of the $B_{1}^{+}$ maps (2nd order, kernel size 9 × 9 × 9). SAR maps derived from experimental $B_{1}^{+}$ maps were then convolved with an averaging filter (kernel size 10 × 10 × 10 mm^3^) in order to provide 1 g averaged SAR maps, similar to those from the simulation. Additionally, 10 g averaged SAR have been calculated to follow standard safety metrics, using an averaging filter with a kernel size of 22 × 22 × 22 mm^3^.

**TABLE 2:** Tissue properties used for experimental SAR map calculation in the phantom and in subjects.

Object	Density (kg.m^−3^)	Relative permittivity	Conductivity (S.m^−1^)	
			$\sigma_{\text{test}}$ (phantom) and $\sigma_{\mathrm{ex}\;\text{vivo}}$ (brain)	$\sigma_{\text{true}}$ (phantom) and $\sigma_{\text{in vivo}}$ (brain)
Phantom	1000	80	0.24	0.47
Brain	1045	63.8	0.46	0.70

SAR maps from the simulation were estimated using the exact definition ${\mathrm{SAR}}_{\text{exact}}=\sigma {\left|E\right|}^{2}/\left(2\rho_{m}\right)$, with E the electric field. They were also estimated using the formula in Equation (4), in order to assess the error solely due to modelling (different geometry and conductivity) and the error due to the $B_{1}^{+}$‐derived SAR approximation formula. Average SAR maps were computed in the region of interest, that is, in the phantom mask, and in the biomodel's or volunteer's brain. Note that all these SAR estimates correspond to the power absorbed by tissues during RF excitation. For practical application, and comparison to the limits of exposure, the RF duty cycle should be taken into account, and a time‐averaged SAR should be calculated.

### 2.5 Comparison of the Total Absorbed Power

The total absorbed power (*P*_abs_) is the measure of the power deposited in a body (all the tissue in the antenna emission field) when exposed to a transmission antenna. It can be calculated (cf. Equation (5)) by subtracting the reflected power (*P*_refl_) and coil power losses (*P*_loss_) from the antenna input power (*P*_input_), which are all monitored during MRI acquisitions. A previous study [13] used this metric, more reliable than the scanner‐reported whole‐body SAR, to compare measurements and simulations on phantoms and patients/human models. For one volunteer, *P*_abs_ was calculated from scanner log file information, and from simulation using the body model which has the closest weight (81 kg for Emma, against 78 kg for the volunteer). The simulated *P*_abs_ was normalized to 11.74 μT [2], which is the averaged $B_{1}^{+}$ magnitude in the central transversal slice, replicating the method from [13]. One simulation was run with the ex vivo conductivity for the brain and a second one with the in vivo conductivity.

(5) $$P_{\mathrm{abs}}=P_{\text{input}}-P_{\text{refl}}-P_{\text{loss}}$$

### 2.6 Statistical Analysis

Wilcoxon signed‐rank tests were used to test whether differences due to changes of the conductivity value or biomodel were significant, both for $B_{1}^{+}$ magnitude/phase maps, and for SAR maps.

## 3 Results

### 3.1 Quantitative Comparison of $B_{1}^{+}$ Maps

**FIGURE 2:**
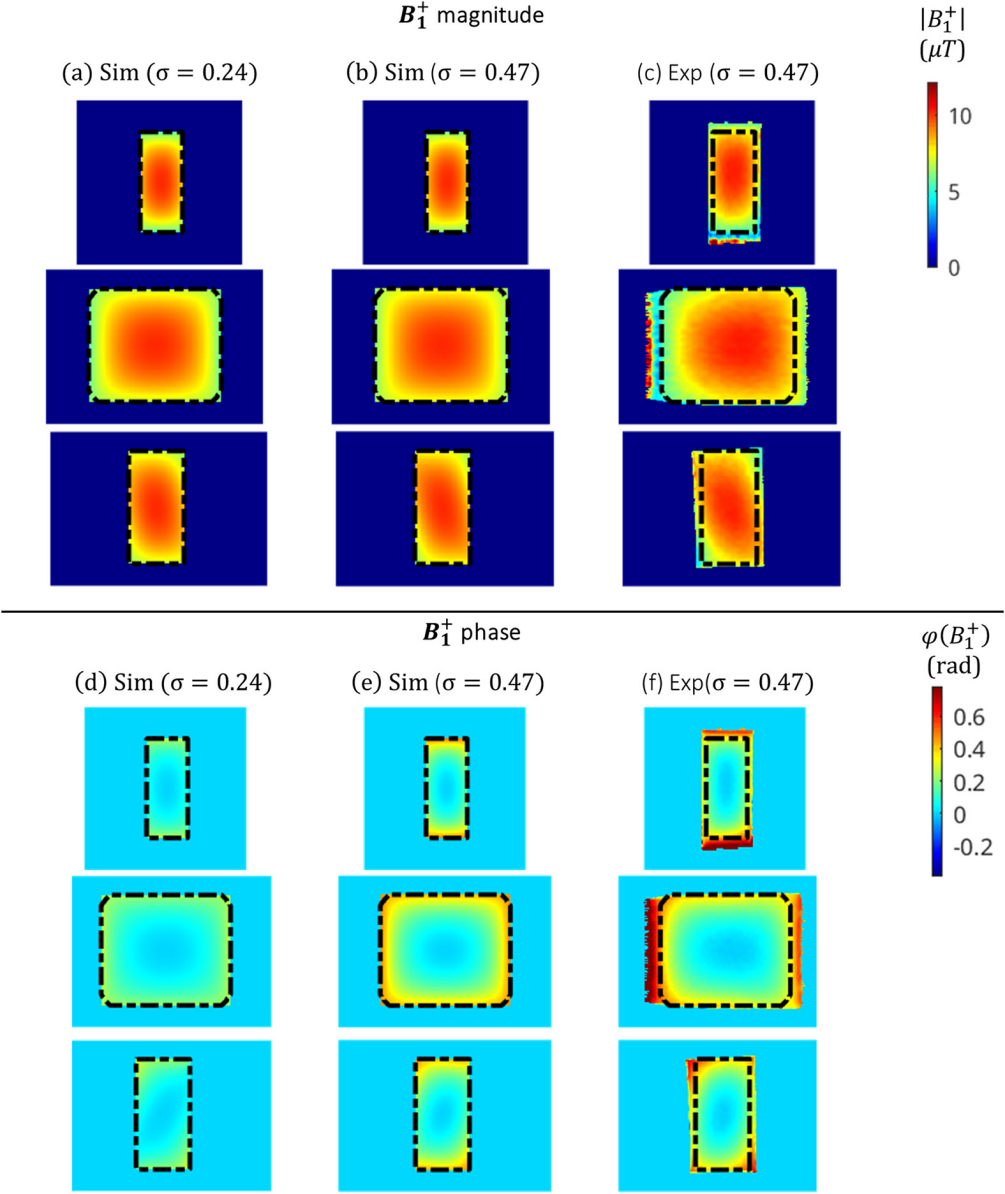
Comparison of simulated and experimental $B_{1}^{+}$ magnitude/phase maps in the phantom experiment (both sagittal and axial planes are shown, the black line is the mask used for quantitative assessment): (a, d) simulation with incorrect conductivity (σ=0.24 S/m); (b, e) simulation with true conductivity (σ=0.47 S/m); (c, f) experimental $B_{1}^{+}$ maps (gel conductivity σ=0.47 S/m).

In the phantom, comparison between simulated $B_{1}^{+}$ maps obtained with the two conductivity values (σ=0.47S/m and σ=0.24S/m) for the gel is shown in Figure 2. Qualitatively, simulated maps seemed to be in good agreement with experimental maps. Small differences can be seen both in magnitude and phase but were more pronounced in the phase. However, the simulation using the true conductivity value (σ=0.47S/m) seemed closer to the experimental $B_{1}^{+}$ map. Quantitative analysis in the phantom region, restricted to the phantom mask at the isocenter, showed that NRMSE in $B_{1}^{+}$, respectively, in magnitude/phase, was 0.09/0.45 with the incorrect conductivity value, and was reduced to 0.08/0.21 with the true conductivity value.

**FIGURE 3:**
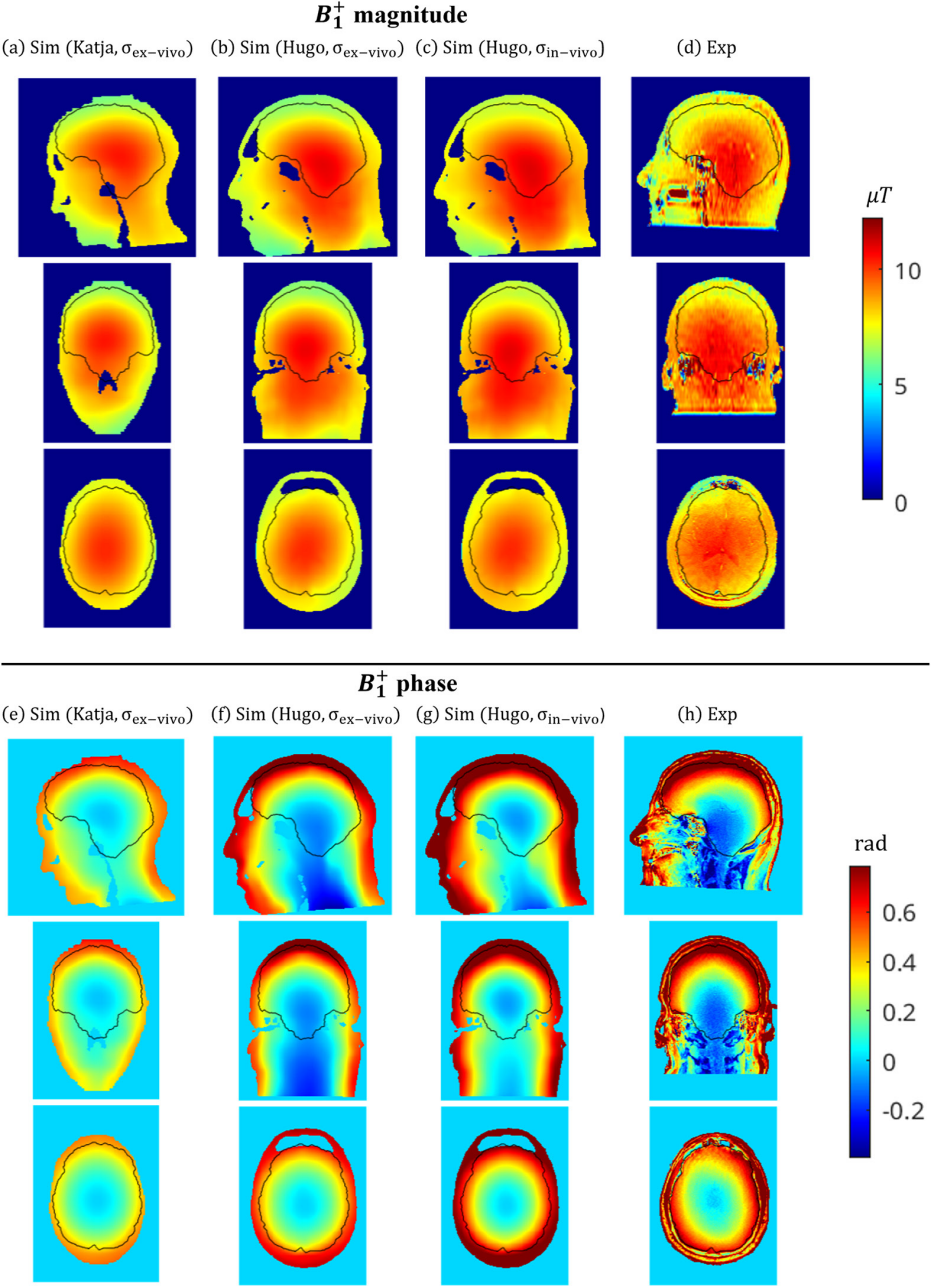
Comparison of simulated and experimental $B_{1}^{+}$ magnitude/phase maps in a healthy subject (sagittal, coronal and axial planes are shown, the black line is the experimental brain contour, segmented from the MPRAGE sequence): (a, e) simulation with the worst biomodel for this subject (Hugo) and the standard brain conductivity ($\sigma_{\mathrm{ex}\;\text{vivo}}=0.46$ S/m); (b, f) simulation with the best biomodel for this subject (Emma) and the standard brain conductivity ($\sigma_{\mathrm{ex}\;\text{vivo}}=0.46$ S/m); (c, g) simulation with the best biomodel for this subject (Emma) and brain conductivity ($\sigma_{\text{in vivo}}=0.70$ S/m); (d, h) experimental $B_{1}^{+}$ maps.

In the subject database, an exemplary comparison between simulated and experimental $B_{1}^{+}$ maps with different biomodels and brain conductivity values is shown in Figure 3. Two other examples can be found in Figures S1 and S3, with Figure S3 corresponding to the worst case of the database in terms of NRMSE. The agreement between simulated and experimental $B_{1}^{+}$ maps seemed to be good, as in the phantom experiment. The choice of the biomodel seemed to impact mostly $B_{1}^{+}$ magnitude. As expected from the literature on MR electrical property mapping, the change of conductivity in the simulation translated into steeper spatial variations in the $B_{1}^{+}$ phase map (conductivity being proportional to the Laplacian of the $B_{1}^{+}$ phase map as a first order approximation [35]), and had no visible impact on $B_{1}^{+}$ magnitude. Each subject was assigned to the biomodel (“Best biomodel”), which had the best NRMSE score when comparing complex $B_{1}^{+}$ maps, conversely, the “Worst biomodel” was the one with the poorest NRMSE score. Quantitative comparison using NRMSE in the brain region is shown in Table 3. A table giving all NRMSE scores for each subject and each biomodel can be found in Table S2. When comparing different biomodels (using the standard brain conductivity $\sigma_{\mathrm{ex}\;\text{vivo}}$), the NRMSE between simulation and experiment in the brain region was improved by 11% for $B_{1}^{+}$ magnitude (0.060 for the worst biomodel and 0.054 for the best one), which was statistically significant (*p* = 0.007), and by 2% for $B_{1}^{+}$ phase (0.370 for the worst and 0.362 for the best), which was not significant (*p* = 0.47). With the best biomodel, using the in vivo brain conductivity value degraded NRMSE by 4% for $B_{1}^{+}$ magnitude (0.056 using $\sigma_{\text{in vivo}}$), which was significant (*p* = 0.04), but improved the NRMSE by 48% for $B_{1}^{+}$ phase (0.221 using $\sigma_{\text{in vivo}}$), which was significant (*p* = 0.0004).

**TABLE 3:** Normalized root‐mean‐squared error (NRMSE) between simulated $B_{1}^{+}$ maps and experimental in $B_{1}^{+}$ maps (16 healthy subjects), in the brain region, using different biomodels and brain conductivity values ($\sigma_{\mathrm{ex}\;\text{vivo}}$ or $\sigma_{\text{in vivo}}$).

	Worst biomodel, $\sigma_{\mathrm{ex}\;\text{vivo}}$	Best biomodel, $\sigma_{\mathrm{ex}\;\text{vivo}}$	*p* (worst vs. best)	Best biomodel, $\sigma_{\text{in vivo}}$	*p* ($\sigma_{\mathrm{ex}\;\text{vivo}}$ vs. $\sigma_{\text{in vivo}}$)
NRMSE in $B_{1}^{+}$ magnitude	0.060 (±0.010)	0.054 (±0.007)	**0.007** *	0.056 (±0.007)	**0.04** *
NRMSE in $B_{1}^{+}$ phase	0.370 (±0.086)	0.362 (±0.051)	0.47	0.221 (±0.031)	**0.0004** *

* Statistically significant difference.

The NRMSE between multiclass and single‐class brain maps are given in Table 4. When comparing the complex $B_{1}^{+}$ maps, NRMSE were 9% for ex vivo conductivities and 0.1% for in vivo conductivities. The low degree of differences validates the chosen approach in using a single brain conductivity in simulations and the postprocessing steps. A brain mask is applied, based on the intersection of Hugo's brain mask and subject 10's one.

**TABLE 4:** Errors in $B_{1}^{+}$ and brain SAR using a single‐class or a multiclass brain model (Hugo vs. subject 10), and either in vivo or ex vivo conductivity values.

Brain simulation type	$B_{1}^{+}$ maps (NRMSE)						1 g‐averaged brain SAR (W/kg)	
	Complex		Magnitude		Phase			
	$\sigma_{\mathrm{ex}\;\text{vivo}}$	$\sigma_{\text{in vivo}}$	$\sigma_{\mathrm{ex}\;\text{vivo}}$	$\sigma_{\text{in vivo}}$	$\sigma_{\mathrm{ex}\;\text{vivo}}$	$\sigma_{\text{in vivo}}$	$\sigma_{\mathrm{ex}\;\text{vivo}}$	$\sigma_{\text{in vivo}}$
Single‐class brain^a^	0.1361	0.0984	0.0658	0.0589	0.2865	0.1840	14.93	19.79
Multiclass brain^b^	0.1489	0.0983	0.0645	0.0588	0.3212	0.1825	15.07	20.03
Relative difference between the two simulations	0.09	0.001	0.02	0.002	0.114	0.008	0.009	0.012

^a^ $\sigma_{\text{whole brain};\mathrm{ex}\;\text{vivo}}=0.46\;S/m;\sigma_{\text{whole brain};\text{in vivo}}=0.70\;S/m.$

^b^ $\sigma_{\mathrm{WM};\mathrm{ex}\;\text{vivo}}=0.34\;S/m,\sigma_{\mathrm{GM};\mathrm{ex}\;\text{vivo}}=0.58\;S/m,\sigma_{\text{Cereb};\mathrm{ex}\;\text{vivo}}=0.82\;S/m$; $\sigma_{\mathrm{WM};\text{in vivo}}=0.66\;S/m,\sigma_{\mathrm{GM};\text{in vivo}}=0.74\;S/m,\sigma_{\text{Cereb};\text{in vivo}}=0.70$ S/m.

### 3.2 Quantitative Assessment of SAR

**FIGURE 4:**
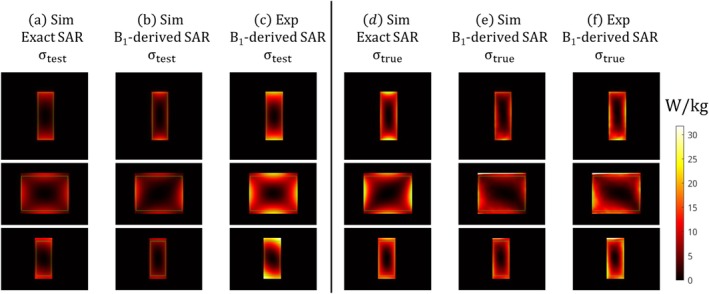
Comparison of SAR maps in the phantom, with different estimation methods (sagittal, coronal and axial planes are shown, the green line is the contour for the global SAR calculation): (a) simulated SAR using exact calculation and test conductivity value $\sigma_{\text{test}}=0.24\;S/m$; (b) simulated SAR using $B_{1}^{+}$‐derived estimation and test conductivity value; (c) experimental SAR using $B_{1}^{+}$‐derived estimation and test conductivity value; (d, e, f) same as (a, b, c) but using brain conductivity $\sigma_{\text{true}}$ = 0.47 S/m.

SAR values for the phantom can be found in Table 5 and SAR maps are shown in Figure 4. For σ=0.24 S/m, the differences between the simulated exact SAR/$B_{1}^{+}$‐derived SAR and the experimental $B_{1}^{+}$‐derived SAR were, respectively, 15%/21% and, for σ=0.47 S/m, they were 4% /4%. These results highlight the good accuracy of the $B_{1}^{+}$‐derived approach for SAR calculation on a homogeneous material, when the conductivity value is properly set in the simulation. Conversely, an incorrect conductivity value can lead to significantly higher errors in SAR estimates.

**TABLE 5:** Specific absorption rate averaged in the brain region (on 16 healthy subjects) and 1 g/10 g‐averaged peak spatial SAR (1 g/10 g‐psSAR, in the phantom/brain region), as computed by simulation (exact calculation or $B_{1}^{+}$ derived estimation) and by experiment ($B_{1}^{+}$ derived estimation), using different brain conductivity values ($\sigma_{\mathrm{ex}\;\text{vivo}}$ or $\sigma_{\text{in vivo}}$).

	Conductivity (S/m)	Simulation, exact SAR calculation [1 g/10 g‐psSAR] (W/kg)^a^	Simulation, $B_{1}^{+}$‐derived SAR estimation [1 g/10 g‐psSAR] (W/kg)^a^	Experiment, $B_{1}^{+}$‐derived SAR estimation [1 g/10 g‐psSAR] (W/kg)^a^
Phantom	0.24	4.7 [11.5/6.8]	4.1 [12.9/7.1]	6.3 [20.7/11.0]
	0.47	9.2 [24.9/13.6]	7.8 [23.6/12.9]	8.5 [27.9/14.8]
Brain	$\sigma_{\mathrm{ex}\;\text{vivo}}=0.46$	11.4 (±2.2) [30.1 ± 9.9/18.0 ± 2.9]	13.2 (±2.5) [33.1 ± 8.6/22.8 ± 4.0]	25.0 (±4.1) [177.5 ± 52.6/39.1 ± 7.9]
	$\sigma_{\text{in vivo}}=0.70$	15.6 (±2.7) [40.6 ± 12.3/25.0 ± 3.6]	18.1 (±2.8) [44.6 ± 8.9/30.8 ± 4.6]	22.5 (±3.7) [160.0 ± 47.4/35.2 ± 7.2]

^a^ SAR values reported here correspond to instantaneous values during RF excitation; for comparison with limits of exposure, time‐averaging for a particular pulse sequence (including pulse shape and effective duty cycle) should be taken into account.

**FIGURE 5:**
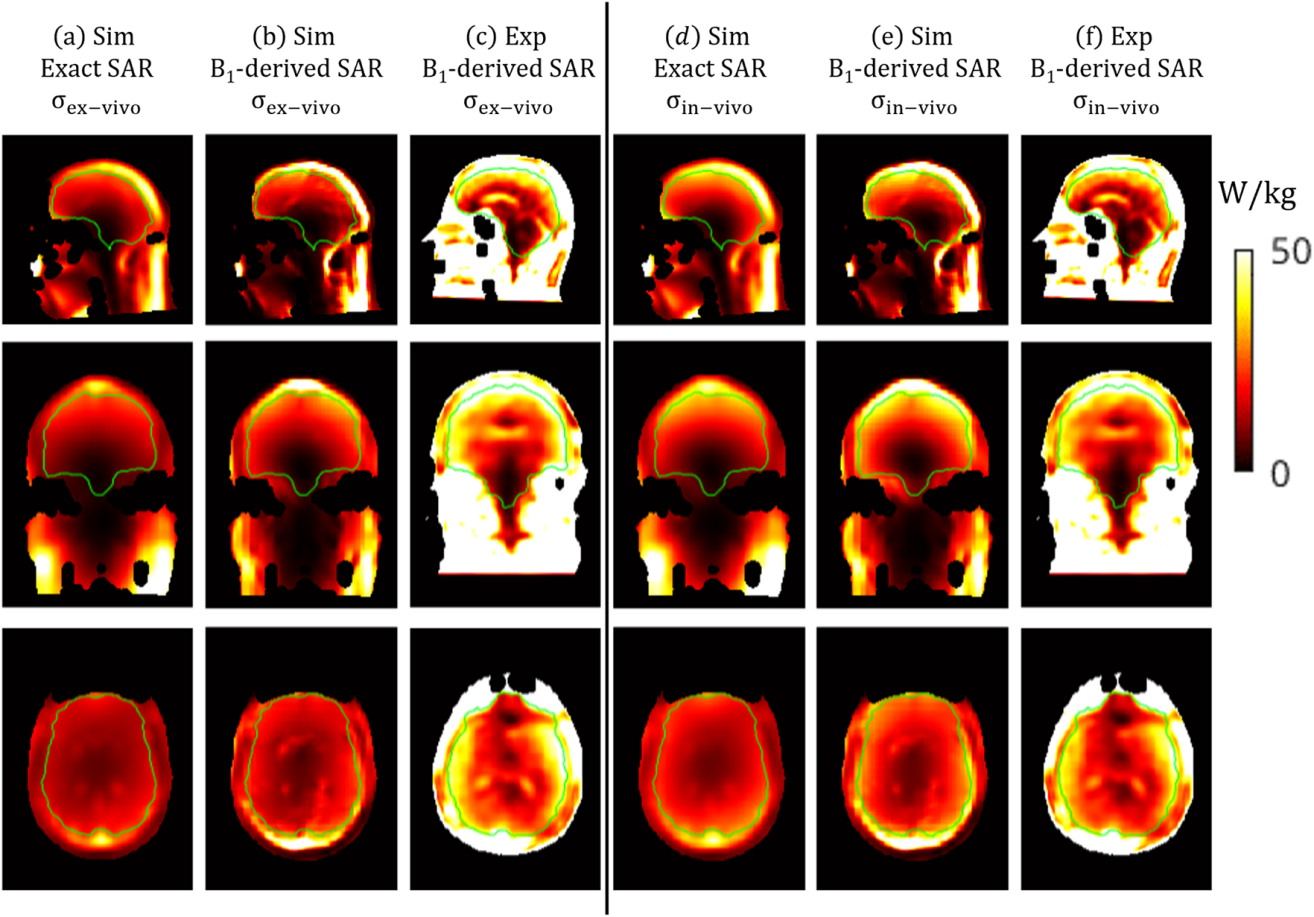
Comparison of SAR maps in a healthy subject, with different estimation methods (sagittal, coronal and axial planes are shown, the green line is the brain contour of the biomodel, in simulated maps, or the subject, in experimental maps): (a) simulated SAR using exact calculation and standard brain conductivity ($\sigma_{\mathrm{ex}\;\text{vivo}}=0.46$ S/m); (b) simulated SAR using $B_{1}^{+}$‐derived estimation and standard brain conductivity; (c) experimental SAR using $B_{1}^{+}$‐derived estimation and standard brain conductivity; (d, e, f) same as (a, b, c) but using brain conductivity $\sigma_{\text{in vivo}}=0.70$ S/m. Here, the biomodel used for simulation was Emma (best match for $B_{1}^{+}$ maps).

Exemplary SAR maps from a subject, obtained with different SAR estimation methods, are shown in Figure 5. The biomodel minimizing NRMSE in complex $B_{1}^{+}$ in the brain was used (Hugo in this case). Another example is shown in Figure S2, and the worst case of the database is shown in Figure S4. Visually, all SAR maps seemed in relatively good agreement in the brain, with brain peak SAR values found at the superior periphery, for example, in the parietal region, and SAR values decreasing toward the cerebellum. However, the experimental SAR maps outside the brain seemed much less reliable, especially in the lower head, which exhibited large regions with elevated SAR values, above 50 W/kg, which were not consistent with the simulated SAR maps.

Quantitative results in the brain region are summarized in Table 5. First, the comparison between simulations with the exact or $B_{1}^{+}$‐derived formula showed that the latter one overestimates the exact SAR calculation by a bit more than 15%. Second, the comparison between simulated and experimental SAR, both estimated with the $B_{1}^{+}$‐derived formula, showed a better agreement when $\sigma_{\text{in vivo}}$ was used as the brain conductivity (18.1 W/kg vs. 22.5 W/kg) rather than $\sigma_{\mathrm{ex}\;\text{vivo}}$ (13.2 W/kg vs. 25.0 W/kg). This improvement was significant (*p* = 0.0004).

Local 1 g‐averaged and 10 g‐averaged peak spatial SAR values are also reported in Table 5. The mismatch between simulation and experimental values are also reduced when using $\sigma_{\text{in vivo}}$, although some overly high values are found in the experimental 1 g‐averaged SAR maps. These high local SAR values corresponded to a small number of voxels located at the periphery of the brain, for example, in the occipital or parietal region in Figure 5f. Despite the brain mask erosion, these values may be less reliable due to the application of the spatial differentiation kernel near the boundary between two different tissues. Furthermore, when using a 10 g average, the peak SAR values are significantly smaller.

We found a measured absorbed power of 973 W using RF power measurements from the scanner recorded in log files. Absorbed power from the simulation was 582 W (60% of the measured value) when using ex vivo conductivity for the brain and 633 W (65% of the measured value) when using in vivo conductivity for the brain. It should be noted that the Emma human model, even though similar in weight with the volunteer investigated, has missing arms (below half of the humerus) and legs (below half of the thigh).

## 4 Discussion

Several contributions presented in this paper may be relevant to the field of MRI safety. First, we proposed a methodological framework for comparing experimental and simulated $B_{1}^{+}$ and SAR maps in the brain. Provided a model of the transmit coil is available, this framework allows for a direct comparison of complex $B_{1}^{+}$ maps. A key step in achieving a good comparison was the method for combining the two excitation ports in the simulation, based on simulated and experimental S‐parameters, to account for differences in load impedance. In other studies [36], such port combination was tuned empirically. In a recent study [37], errors between simulated and experimental $B_{1}^{+}$ maps have been investigated on two subjects. For each one, a specific anatomical head was generated, and a generic birdcage coil model was used for all the simulations. The $B_{1}^{+}$ maps errors are given for the head and only the error on magnitude was given, the metric used was the mean absolute percentage error (MAPE): 6.63% (subject 1) and 5.16% (subject 2).

Another contribution is the new formula, given in Equation (4), for $B_{1}^{+}$‐derived SAR estimation. This formula was obtained using a rational similar to that in [38] with fewer terms being neglected. Although it overestimated exact SAR by approximately 15% in the subjects, it is slightly more general than another formula proposed by Voigt et al. [27], which was the same as Equation (4) without the z‐derivative term. It should be recalled that, in order to translate the brain SAR estimates presented here into a value that can be compared with SAR limits given by international standards (normal mode: 2 W/kg for the whole body, 3.2 W/kg for the head [39]), it should be averaged in time, considering the duty cycle of a particular MRI pulse sequence.

In the phantom experiments, it has been observed that the wider the phantom the poorer the results when applying this framework for comparing both complex $B_{1}^{+}$ and SAR maps. To tackle this issue, we used a phantom with dimensions comparable to those of a human head. This is significantly smaller than conventionally used ASTM phantoms, where errors would be larger. In the subjects, after scaling the simulated fields and optimizing for the biomodel and brain conductivity, the error in the brain (NRMSE) was 5.6% in $B_{1}^{+}$ magnitude and 22.1% in $B_{1}^{+}$ phase. Unlike $B_{1}^{+}$, for in vivo SAR values, there was no ground truth. However, we were able to estimate the error in $B_{1}^{+}$‐derived SAR maps between simulation and experiment (approximately 15%), which can be attributed to errors in the modelling, including the field source (transmit coil) and the medium (biomodel and electrical properties). In particular, no gradient coils, magnet, or patient table was modelled in our simulation. These elements may modify the distribution of the RF fields (and thereby SAR) or the impedance seen by the coil (i.e., the S parameters). We also estimated the error between $B_{1}^{+}$‐derived SAR and exact SAR, which was between 8% and 15%. This error can be attributed to terms that were neglected to derive Equation (4).

From our results when working on the absorbed power and from the recent study by Paysen et al. [13], there are major disparities between measurements and simulations in human subjects. It demonstrates that further work is needed to fully understand causes of such mismatches with human data. Possible explanations include incorrect electrical property values used for human tissues, limitations of power measurements/global SAR calculation from MRI systems, approximations from simulation software (modeling/monitoring of antenna, solver types, and mesh definition …). Although brain represents less than 2% of total body mass, the suggested change in conductivity value accounted for more than 5% of whole body SAR.

Our results also suggest that the brain conductivity value that is commonly used in electromagnetic simulation may need to be revised. This claim is supported by the reduced error between simulated and experimental $B_{1}^{+}$ maps, mostly in the phase, as well as the better agreement in average brain SAR, when correcting for brain conductivity. The commonly used conductivity values come from the extensive work by Gabriel et al. [14] that allowed the first databases of tissue electrical properties to be created and made available [33]. These conductivity values, for the brain, were obtained by probe measurements from samples of a single ex vivo human cadaver. However, complementary information about the cadaver was lacking, such as age, sex, height, weight, medical condition, and so forth. While in vivo electrical property mapping is still a recent technique that needs more validation studies, as demonstrated in [18, 20], the study by He et al. [16] on 17 adult subjects showed consistent, reproducible brain conductivity values of 0.74 ± 0.07 S/m in the gray matter, and 0.66 ± 0.04 S/m in the white matter, in healthy adults aged from 25 to 73 years old, with body mass indexes from 19.6 to 32.6 kg/m^2^. These were significantly higher than the commonly used database at 128 MHz, which gives: 0.58 S/m for gray matter and 0.34 S/m for white matter.

The impact of this increased conductivity on SAR may be surprising, since the experimentally estimated SAR was decreased. One might expect SAR to simply increase linearly with conductivity when considering the exact SAR formula ($\sigma {\left|E\right|}^{2}/\left(2\rho_{m}\right)$). However, a change of conductivity also affects the **E** field distribution. In the $B_{1}^{+}$‐derived formula (Equation (4)), conductivity also appears in the denominator within the κ coefficient, which is squared, thereby explaining the decrease in SAR.

Accurate $B_{1}^{+}$ mapping is required to apply the proposed framework. Here, the vendor's presaturated turbo FLASH sequence was used for magnitude $B_{1}^{+}$ mapping [29]. This method has the advantage of being fast, but it is a 2D multislice acquisition with a relatively large slice gap. Alternative sequences can be used, such as actual flip angle imaging (AFI) [40], Bloch Siegert shift [41], or DREAM [42]. For $B_{1}^{+}$ phase mapping, the proposed UTE sequence has the advantage of being a 3D high resolution scan, relatively robust to $B_{0}$ inhomogeneities due to the short echo time. Alternative sequences could be used, such as a 2D multislice spin echo or fast spin echo, or a 3D phase‐cycled balanced steady‐state free precession [43]. It should be kept in mind that all these sequences provide an estimate of the transceive phase map ($\varphi_{\mathrm{TR}}$), which is the sum of the transmission phase ($\varphi_{+}$) and the reception phase ($\varphi_{-}$). The transceive phase assumption that we used, that is, $\varphi_{+}=\varphi_{-}=\varphi_{\mathrm{TR}}/2$, is generally considered to be valid at field strengths of 3T or lower [30].

There are several limitations in the present study. An averaged conductivity value was used for the whole brain (gray and white matter) due to the limitations of our biomodel family. We checked that this simplified single‐class brain model did not change the results significantly compared to a multiclass one (with separate classes for WM and GM), both in terms of $B_{1}^{+}$ maps and brain SAR values. The residual errors may be explained by the geometric mismatch between the biomodel and the actual subject, the residual misalignment between them (only translations were corrected, but rotations were also observed in our database). In the SAR maps shown in Figure 5, SAR values outside the brain were not consistent with simulation. This can be explained by the insufficient accuracy of the spatial derivative calculation in Equation (4). We chose to use a relatively narrow kernel for the Savitzky–Golay filter to avoid oversmoothing the resulting SAR maps, so that 1 g‐averaged SAR maps can be estimated. Alternatively, a wider kernel could have been used, in combination with 10 g‐averaged SAR maps, and a wider erosion of the brain mask to avoid boundary effects. Another source of inaccuracy is that, for simplicity, we used brain properties (conductivity, permittivity, and mass density) in the whole head to generate the $B_{1}^{+}$‐derived SAR map. For this reason, we only calculated quantitative SAR values in the brain mask.

## 5 Conclusions

A framework has been proposed to quantitatively evaluate the accuracy of electromagnetic simulation using human biomodels, for MRI safety applications at 3T. It allowed the simulated $B_{1}^{+}$ fields from a two‐port transmit coil model to be optimally combined, aligned, and scaled, so that they can be directly compared to complex $B_{1}^{+}$ maps acquired by MRI. A quantitative comparison of simulated and experimental SAR maps was also proposed, using different estimation methods, including a novel $B_{1}^{+}$‐derived formula. In vivo comparison in a small database of healthy adults, with various biomodels, showed that the electrical conductivity of the brain has a significant impact on $B_{1}^{+}$ distribution in the brain, especially in the $B_{1}^{+}$ phase, and in the subsequent SAR estimation. Finally, a better agreement was found between simulation and experiment when using in vivo brain conductivity values, obtained from recent MR electrical property mapping studies, rather than the commonly used literature values from ex vivo studies.

## Author Contributions

M.R. imaging protocol: G.P., Z.H., F.O.; electromagnetic simulation: G.P., G.R., F.O.; processing of the B1+ fields: G.P., Z.H., K.A., P.S., F.O.; voxel‐wise quantitative comparison and statistical analysis: G.P., Z.H., P.F., P.S., F.O.; writing – original draft preparation: G.P., F.O.; writing – review and editing: Z.H., P.F, F.O.; supervision: J.F., F.O.; project administration: F.O. All authors have read and agreed to the published version of the manuscript.

## Funding

This work was funded by Grant Number ANR‐21‐ce19‐0040 (ELECTRA project). This study also received support from CPER IT2MP and FEDER (European Regional Development Fund). This work was performed on a platform member of France Life Imaging network (grant ANR‐11‐INBS‐0006).

## Conflicts of Interest

Dr. Grecia Romero and Dr. Pauline Ferry are employees of Healtis Company. Dr. Khalid Ambarki is an employee of Siemens Healthcare Company. Pr. Jacques Felblinger is a shareholder of Healtis Company.

## Supporting information


**Table S1:** Main characteristics for all the subjects included in the study.
**Table S2:** Normalized root‐mean‐squared error (NRMSE) between simulated $B_{1}^{+}$ maps and experimental $B_{1}^{+}$ maps for 16 healthy subjects, in the brain region, using different biomodels (Emma, Katja, and Hugo) and brain conductivity values ($\sigma_{\mathrm{ex}\;\text{vivo}}$ or $\sigma_{\text{in vivo}}$). For each subject, the biomodel selected as “best biomodel” is in bold and was selected based on its NRMSE complex score.
**Figure S1:** Comparison of simulated and experimental $B_{1}^{+}$ magnitude/phase maps in subject 6 (sagittal, coronal and axial planes are shown, the black line is the experimental brain contour, segmented from the MPRAGE sequence): (a, e) simulation with another biomodel for this subject (Katja) and the standard brain conductivity ($\sigma_{\mathrm{ex}\;\text{vivo}}=0.46$ S/m); (b, f) simulation with the best biomodel for this subject (Emma) and the standard brain conductivity ($\sigma_{\mathrm{ex}\;\text{vivo}}=0.46$ S/m); (c, g) simulation with the best biomodel for this subject (Emma) and brain conductivity ($\sigma_{\text{in vivo}}=0.70$ S/m); (d, h) experimental $B_{1}^{+}$ maps.
**Figure S2:** Comparison of SAR maps in subject 6, with different estimation methods (sagittal, coronal and axial planes are shown, the green line is the brain contour of the biomodel, in simulated maps, or the subject, in experimental maps): (a) simulated SAR using exact calculation and standard brain conductivity ($\sigma_{\mathrm{ex}\;\text{vivo}}=0.46$ S/m); (b) simulated SAR using $B_{1}^{+}$‐derived estimation and standard brain conductivity; (c) experimental SAR using $B_{1}^{+}$‐derived estimation and standard brain conductivity; (d, e, f) same as (a, b, c) but using brain conductivity $\sigma_{\text{in vivo}}=0.70$ S/m. Here, the biomodel used for simulation was Emma (best match for $B_{1}^{+}$ maps).
**Figure S3:** Comparison of simulated and experimental $B_{1}^{+}$ magnitude/phase maps in the subject with the highest NRMSE score for complex $B_{1}^{+}$ maps (subject 14). The NRMSE scores are 0.22 for $\sigma_{\mathrm{ex}\;\text{vivo}}$ complex map and 0.15 for $\sigma_{\text{in vivo}}$ complex map. The mean NRMSE scores on all the subjects and their respective best fitting biomodel are 0.14 ± 0.03 for $\sigma_{\mathrm{ex}\;\text{vivo}}$ complex maps and 0.10 ± 0.02 for $\sigma_{\text{in vivo}}$ complex maps. Sagittal, coronal, and axial planes are shown, the black line is the experimental brain contour, segmented from the MPRAGE sequence: (a, e) simulation with another biomodel for this subject (Katja) and the standard brain conductivity ($\sigma_{\mathrm{ex}\;\text{vivo}}=0.46$ S/m); (b, f) simulation with the best biomodel for this subject (Hugo) and the standard brain conductivity ($\sigma_{\mathrm{ex}\;\text{vivo}}=0.46$ S/m); (c, g) simulation with the best biomodel for this subject (Hugo) and brain conductivity ($\sigma_{\text{in vivo}}=0.70$ S/m); (d, h) experimental $B_{1}^{+}$ maps.
**Figure S4:** Comparison of SAR maps in the subject with the highest NRMSE score for complex $B_{1}^{+}$ maps (subject 14), with different estimation methods (sagittal, coronal and axial planes are shown, the green line is the brain contour of the biomodel, in simulated maps, or the subject, in experimental maps): (a) simulated SAR using exact calculation and standard brain conductivity ($\sigma_{\mathrm{ex}\;\text{vivo}}=0.46$ S/m); (b) simulated SAR using $B_{1}^{+}$‐derived estimation and standard brain conductivity; (c) experimental SAR using $B_{1}^{+}$‐derived estimation and standard brain conductivity; (d, e, f) same as (a, b, c) but using brain conductivity $\sigma_{\text{in vivo}}=0.70$ S/m. Here, the biomodel used for simulation was Hugo (best match for $B_{1}^{+}$ maps).

## Data Availability

The data that support the findings of this study are available on request from the corresponding author. The data are not publicly available due to privacy or ethical restrictions.

## Appendix 1 Combination of Excitation Ports in the Simulation

When only one port is considered in a RF simulation of a birdcage coil, and the capacitance is tuned to be close to resonance, the difference between the simulated and experimental S‐parameters does not affect the electromagnetic field distribution (both are the same, up to a complex factor) [44]. On the other hand, the difference between experimental and simulated S‐parameters, when combining the fields from two distinct ports (as it is generally the case for the whole‐body transmit birdcage coil of 3T MRI systems), does influence the field distribution, since the complex ratio of RF voltages actually transmitted to the antenna determines this distribution.

The relationship between voltage $V_{j,\mathrm{app}}$ applied to port j and voltage $V_{j,\mathrm{ant}}$ actually delivered to the antenna (i.e., the voltage at port *j* of the antenna, not the voltage transmitted though the antenna) is

(A1)
$$V_{j,\mathrm{ant}}=V_{j,\mathrm{app}}\frac{Z_{j}}{Z_{0}+Z_{j}}$$

where $Z_{j}$ is the input impedance at port *j* and $Z_{0}=50\;\Omega$. The input impedances $Z_{j}$ can be deduced from the S‐parameters using the following formula:

(A2)
$$Z_{j}=\frac{1+S_{\mathrm{jj}}}{1-S_{\mathrm{jj}}}Z_{0}$$

For the ratio between simulated and experimental transmitted voltages to be the same, the following condition must be fulfilled:

(A3)
$$\frac{V_{2,\mathrm{tr}}^{\mathrm{sim}}}{V_{1,\mathrm{ant}}^{\mathrm{sim}}}=\frac{V_{2,\mathrm{tr}}^{\exp}}{V_{1,\mathrm{ant}}^{\exp}}$$

Using experimental/simulated applied voltages and input impedances:

(A4)
$$\frac{V_{2,\mathrm{app}}^{\mathrm{sim}}}{V_{1,\mathrm{app}}^{\mathrm{sim}}}=\frac{V_{2,\mathrm{app}}^{\exp}}{V_{1,\mathrm{app}}^{\exp}}R$$

with R the correction factor calculated from the input impedances, which can be easily derived from S‐parameters from Equation (A2):

(A5)
$$R=\frac{Z_{2}^{\exp}}{\left(Z_{2}^{\exp}+Z_{0}\right)}\frac{\left({\mathrm{ZZ}}_{1}^{\exp}+Z_{0}\right)}{Z1_{\exp}}\frac{\left(Z_{2}^{\mathrm{sim}}+{\mathrm{ZZ}}_{0}\right)}{Z_{2}^{\mathrm{sim}}}\frac{Z_{1}^{\mathrm{sim}}}{\left(Z_{1}^{\mathrm{sim}}+Z_{0}\right)}$$

$Z_{1}^{\exp}$ and $Z_{2}^{\exp}$ are obtained according to Equation (A6) from the MRI scanner S‐parameters (*S*_11_ and *S*_22_) measured during acquisition and stored within the private DICOM field (0021, 1019) (searching for “ScatterMatrix”).

(A6)
$$Z_{n}^{\exp}=Z_{0}\times \frac{1+S_{\mathrm{nn}}}{1-S_{\mathrm{nn}}}\;\text{with}\;n\;\epsilon \;\left\{1;2\right\}$$

## Appendix 2 $B_{1}^{+}$‐Derived SAR Approximation

Local SAR [26] can be expressed as a function of the electric field **E** or the magnetic field **B** using Maxwell‐Ampere's law:

(A7)
$$\mathrm{SAR}=\frac{\sigma {\left|E\right|}^{2}}{2\rho_{m}}=\frac{\sigma \;}{2\rho_{m}{\left|\mu_{0}\kappa \right|}^{2}}{\left|\nabla \times B\right|}^{2}$$

where $\rho_{m}$ is the tissue mass density and $\kappa =\sigma +\mathrm{i\omega}\varepsilon_{0}\varepsilon_{r}$ is the admittivity.

The magnetic field B can be further related to the circular field components $B_{1}^{+}≝\frac{1}{2}\left(B_{x}+iB_{y}\right)$ and $B_{1}^{-}≝\frac{1}{2}\left(B_{x}-iB_{y}\right)$ of the transmit birdcage coil [45]:

(A8)
$$B=\left[\begin{array}{c} B_{x}\\ B_{y}\\ B_{z} \end{array}\right]=\left[\begin{array}{c} B_{1}^{+}+B_{1}^{-}\\ -i\left(B_{1}^{+}-B_{1}^{-}\right)\\ B_{z} \end{array}\right]$$

Under the commonly adopted approximation $\nabla B_{z}\approx 0$, which is valid for birdcage/quadrature transmit coils in the imaging center, the curl of **B** can be expressed as follows:

(A9)
$$\nabla \times B=\left[\begin{array}{c} \frac{\partial B_{z}}{\partial y}-\frac{\partial B_{y}}{\partial z}\\ \frac{\partial B_{x}}{\partial z}-\frac{\partial B_{z}}{\partial x}\\ \frac{\partial B_{y}}{\partial x}-\frac{\partial B_{x}}{\partial y} \end{array}\right]\approx \left[\begin{array}{c} -\frac{\partial B_{y}}{\partial z}\\ \frac{\partial B_{x}}{\partial z}\\ \frac{\partial B_{y}}{\partial x}-\frac{\partial B_{x}}{\partial y} \end{array}\right]$$

In practice, only $|B_{1}^{+}|$ and the transceive phase $\phi_{\mathrm{tr}}$ are measurable in MRI. Therefore, SAR estimation requires additional assumptions regarding the unknown $B_{1}^{-}$, together with the common transceive phase approximation $\phi^{+}\approx \phi^{-}\approx \phi_{\mathrm{tr}}/2$. Different $B_{1}^{+}$‐based SAR formulations thus arise from how $B_{1}^{-}$ is modeled, as detailed out in the following.

1 $B_{1}^{-}$ **‐excluded SAR** [27, 37, 46]

A widely used approximation neglects $B_{1}^{-}$ entirely, setting

(A10)
$$B_{x}=B_{1}^{+},B_{y}=-{\mathrm{iB}}_{1}^{+}.$$

Substituting into (A7) yields

(A11)
$${\mathrm{SAR}}_{B_{1}^{-}-\text{excluded}}=\frac{\sigma \;}{2\rho_{m}{\left|\mu_{0}\kappa \right|}^{2}}\left(2{\left|\frac{\partial B_{1}^{+}}{\partial z}\right|}^{2}+{\left|\frac{\partial B_{1}^{+}}{\partial x}-i\frac{\partial B_{1}^{+}}{\partial y}\right|}^{2}\right)$$

Although practical, this formulation systematically underestimates SAR, as contributions of $B_{1}^{-}$ to the spatial derivatives of the magnetic field are ignored. Recent subject‐specific validation studies [37, 47] have reported correction factors ranging from approximately 1.79–3.08, depending on anatomy.

2 $E_{z}$ **‐only SAR** [48, 49]

An alternative strategy assumes that $B_{1}^{+}$ and $B_{1}^{-}$ contribute equally to the transverse magnetic field components (valid if $\nabla B_{z}\approx 0$), yielding

(A12)
$$B_{x}=2B_{1}^{+},B_{y}=-2{\mathrm{iB}}_{1}^{+}.$$

The $E_{z}$‐only SAR approximation further assumes that the longitudinal electric field $E_{z}$ dominates local SAR:

(A13)
$$|E_{z}|\gg |E_{x}|,|E_{y}|$$

This leads to

(A14)
$${\mathrm{SAR}}_{E_{z}-\text{only}}=\frac{2\sigma \;}{\rho_{m}{\left|\mu_{0}\kappa \right|}^{2}}\left({\left|\frac{\partial B_{1}^{+}}{\partial x}-i\frac{\partial B_{1}^{+}}{\partial y}\right|}^{2}\right)$$

However, this approximation implicitly suppresses all SAR contribution arising from longitudinal field variation:

(A15)
$$\frac{\partial B_{x,y}}{\partial z}\approx 0,\;i.e.,\frac{\partial B_{1}^{+}}{\partial z}\approx 0$$

While this may hold near the central transverse plane of symmetric birdcage/quadrature coils, it becomes increasingly violated (underestimated) away from this plane, where longitudinal field gradients are nonnegligible [26].

3 **Symmetry‐informed** $B_{1}^{+}$ **‐derived SAR formulation (proposed)**

Here we adopt a symmetry‐informed approach without neglecting any field. Under left–right mirror symmetry at the imaging center and ideal quadrature excitation [45], we assume

(A16)
$$\left|B_{1}^{+}\left(x,y,z\right)\right|\approx \left|B_{1}^{-}\left(-x,y,z\right)\right|$$

with characteristic spatial gradient patterns:

(A17)
$$\frac{\partial B_{1}^{+}}{\partial x}\approx -\frac{\partial B_{1}^{-}}{\partial x},\;\frac{\partial B_{1}^{+}}{\partial y}\approx \frac{\partial B_{1}^{-}}{\partial y}\;\text{and}\;\frac{\partial B_{1}^{+}}{\partial z}\approx \frac{\partial B_{1}^{-}}{\partial z}$$

Using these relations, the electric field components simplify to

(A18)
$$E_{x}\propto i\frac{\partial B_{1}^{+}}{\partial z}-i\frac{\partial B_{1}^{-}}{\partial z}\approx 0$$

(A19)
$$E_{y}\propto \frac{\partial B_{1}^{+}}{\partial z}+\frac{\partial B_{1}^{-}}{\partial z}\approx 2\frac{\partial B_{1}^{+}}{\partial z}$$

(A20)
$$\begin{array}{c} E_{z}\propto -i\frac{\partial B_{1}^{+}}{\partial x}+i\frac{\partial B_{1}^{-}}{\partial x}-\frac{\partial B_{1}^{+}}{\partial y}-\frac{\partial B_{1}^{-}}{\partial y}\approx -2\left(i\frac{\partial B_{1}^{+}}{\partial x}+\frac{\partial B_{1}^{+}}{\partial y}\right)\\ =-2i\left(\frac{\partial B_{1}^{+}}{\partial x}-i\frac{\partial B_{1}^{+}}{\partial y}\right) \end{array}$$

The resulting SAR expression is therefore

(A21)
$${\mathrm{SAR}}_{B_{1}^{+}-\text{derived}}=\frac{2\sigma \;}{\rho_{m}{\left|\mu_{0}\kappa \right|}^{2}}\left({\left|\frac{\partial B_{1}^{+}}{\partial z}\right|}^{2}+{\left|\frac{\partial B_{1}^{+}}{\partial x}-i\frac{\partial B_{1}^{+}}{\partial y}\right|}^{2}\right)$$

Note: if $\frac{\partial B_{1}^{+}}{\partial z}\approx 0$, (A21) reduces to $E_{z}$‐only SAR formula (A14).

4 **Quantitative comparison**

In a corresponding study which will be published separately, using simulated brain data, generated with Sim4Life (ZMT Zurich Medtech AG, Switzerland), with the ADEPT brain model and a generic MR birdcage coil model, the three estimation methods were compared quantitatively. These three SAR formulations can be summarized as

$${\mathrm{SAR}}_{B_{1}^{-}-\text{excluded}}<{\mathrm{SAR}}_{E_{z}-\text{only}}\leq {\mathrm{SAR}}_{B_{1}^{+}-\text{derived}}$$

Thus, the proposed symmetry‐informed $B_{1}^{+}$‐derived SAR provides a more complete and generally higher estimate than the previous approximations, as it accounts for both transverse and longitudinal field variations while respecting physical symmetry constraints. In that simulation study, the proposed method showed slightly conservative estimates, overestimating exact SAR by approximately 30%.
