# Supplementary material for: Improved Radiofrequency Safety Modelling in MRI Using In Vivo Measurements of Brain Conductivity
Source: NMR Biomed. 2026 Jun 23;39(8):e70335. doi: 10.1002/nbm.70335 (PMC13291641; doi:10.1002/nbm.70335)

**SUPPLEMENTARY MATERIALS**

**Table S1.** Main characteristics for all the subjects included in the study.

| Subject | Sex | Age (year) | Size (m) | Weight (kg) | BMI (kg/m²) |
| --- | --- | --- | --- | --- | --- |
| Subject 1 | M | 27 | 1.72 | 63 | 21.3 |
| Subject 2 | F | 25 | 1.6 | 69 | 27.0 |
| Subject 3 | M | 42 | 1.82 | 76 | 22.9 |
| Subject 4 | M | 47 | 1.81 | 78 | 23.8 |
| Subject 5 | F | 27 | 1.55 | 47 | 19.6 |
| Subject 6 | F | 27 | 1.7 | 63 | 21.8 |
| Subject 7 | F | 64 | 1.72 | 77 | 26.0 |
| Subject 8 | F | 28 | 1.66 | 67 | 24.3 |
| Subject 9 | F | 25 | 1.5 | 68 | 30.2 |
| Subject 10 | M | 54 | 1.76 | 101 | 32.6 |
| Subject 11 | M | 23 | 1.83 | 85 | 25.4 |
| Subject 12 | F | 72 | 1.56 | 55 | 22.6 |
| Subject 13 | M | 73 | 1.69 | 60 | 21.0 |
| Subject 14 | M | 67 | 1.73 | 83 | 27.7 |
| Subject 15 | M | 71 | 1.86 | 82 | 23.7 |
| Subject 16 | M | 70 | 1.77 | 78 | 24.9 |
| Mean value |  | 46.4 | 1.71 | 72 | 24.68 |
| Standard deviation |  | 18.4 | 0.08 | 10.5 | 2.63 |

**Table S2.** Normalized root-mean-squared error (NRMSE) between simulated $B_{1}^{+}$ maps and experimental $B_{1}^{+}$maps for 16 healthy subjects, in the brain region, using different biomodels (Emma, Katja and Hugo) and brain conductivity values ($\sigma_{ex-vivo}$ or $\sigma_{in-vivo}$). For each subject, the biomodel selected as “Best biomodel” is in bold and was selected based on its NRMSE complex score.

| Subject’s number | Biomodel investigated | $B_{1}^{+}$ map | | | | | |  |
| --- | --- | --- | --- | --- | --- | --- | --- | --- |
|  |  | Complex | | Magnitude | | Phase | |  |
|  |  | $\sigma_{ex-vivo}$ | $\sigma_{in-vivo}$ | $\sigma_{ex-vivo}$ | $\sigma_{in-vivo}$ | $\sigma_{ex-vivo}$ | $\sigma_{in-vivo}$ | |
| Subject 1 | Hugo | 0.11 | 0.09 | 0.05 | 0.06 | 0.27 | 0.21 | |
|  | Emma | 0.14 | 0.10 | 0.05 | 0.06 | 0.37 | 0.21 | |
|  | **Katja** | **0.14** | **0.09** | **0.05** | **0.06** | **0.37** | **0.20** | |
| Subject 2 | Hugo | 0.09 | 0.10 | 0.05 | 0.06 | 0.22 | 0.22 | |
|  | **Emma** | **0.12** | **0.10** | **0.05** | **0.06** | **0.34** | **0.20** | |
|  | Katja | 0.14 | 0.11 | 0.06 | 0.07 | 0.37 | 0.25 | |
| Subject 3 | Hugo | 0.14 | 0.11 | 0.05 | 0.05 | 0.34 | 0.28 | |
|  | Emma | 0.16 | 0.12 | 0.05 | 0.05 | 0.43 | 0.30 | |
|  | **Katja** | **0.15** | **0.11** | **0.05** | **0.05** | **0.41** | **0.26** | |
| Subject 4 | Hugo | 0.13 | 0.11 | 0.07 | 0.07 | 0.28 | 0.25 | |
|  | Emma | 0.15 | 0.11 | 0.06 | 0.06 | 0.39 | 0.26 | |
|  | **Katja** | **0.15** | **0.11** | **0.06** | **0.06** | **0.39** | **0.24** | |
| Subject 5 | Hugo | 0.12 | 0.14 | 0.08 | 0.08 | 0.29 | 0.39 | |
|  | Emma | 0.11 | 0.10 | 0.06 | 0.06 | 0.29 | 0.25 | |
|  | **Katja** | **0.10** | **0.09** | **0.06** | **0.06** | **0.28** | **0.21** | |
| Subject 6 | Hugo | 0.12 | 0.11 | 0.06 | 0.07 | 0.28 | 0.26 | |
|  | **Emma** | **0.13** | **0.09** | **0.05** | **0.05** | **0.34** | **0.19** | |
|  | Katja | 0.13 | 0.09 | 0.05 | 0.06 | 0.36 | 0.20 | |
| Subject 7 | Hugo | 0.17 | 0.13 | 0.08 | 0.09 | 0.37 | 0.23 | |
|  | Emma | 0.19 | 0.13 | 0.07 | 0.08 | 0.44 | 0.25 | |
|  | **Katja** | **0.19** | **0.12** | **0.06** | **0.07** | **0.44** | **0.24** | |
| Subject 8 | Hugo | 0.11 | 0.10 | 0.06 | 0.07 | 0.26 | 0.23 | |
|  | **Emma** | **0.13** | **0.10** | **0.06** | **0.06** | **0.35** | **0.22** | |
|  | Katja | 0.14 | 0.11 | 0.06 | 0.06 | 0.38 | 0.24 | |
| Subject 9 | Hugo | 0.13 | 0.11 | 0.07 | 0.07 | 0.31 | 0.27 | |
|  | Emma | 0.15 | 0.10 | 0.06 | 0.06 | 0.38 | 0.23 | |
|  | **Katja** | **0.14** | **0.09** | **0.05** | **0.06** | **0.37** | **0.20** | |
| Subject 10 | **Hugo** | **0.13** | **0.09** | **0.06** | **0.06** | **0.29** | **0.18** | |
|  | Emma | 0.17 | 0.12 | 0.06 | 0.06 | 0.42 | 0.26 | |
|  | Katja | 0.17 | 0.12 | 0.06 | 0.06 | 0.43 | 0.27 | |
| Subject 11 | **Hugo** | **0.12** | **0.10** | **0.05** | **0.06** | **0.28** | **0.23** | |
|  | Emma | 0.15 | 0.10 | 0.05 | 0.06 | 0.38 | 0.23 | |
|  | Katja | 0.15 | 0.10 | 0.05 | 0.06 | 0.38 | 0.23 | |
| Subject 12 | Hugo | 0.11 | 0.09 | 0.04 | 0.05 | 0.27 | 0.22 | |
|  | **Emma** | **0.13** | **0.08** | **0.04** | **0.05** | **0.35** | **0.17** | |
|  | Katja | 0.13 | 0.08 | 0.05 | 0.05 | 0.36 | 0.18 | |
| Subject 13 | Hugo | 0.09 | 0.10 | 0.05 | 0.05 | 0.24 | 0.28 | |
|  | Emma | 0.12 | 0.10 | 0.05 | 0.05 | 0.33 | 0.25 | |
|  | **Katja** | **0.12** | **0.10** | **0.05** | **0.05** | **0.34** | **0.24** | |
| Subject 14 | **Hugo** | **0.22** | **0.15** | **0.04** | **0.04** | **0.45** | **0.30** | |
|  | Emma | 0.26 | 0.20 | 0.06 | 0.06 | 0.55 | 0.40 | |
|  | Katja | 0.25 | 0.19 | 0.06 | 0.06 | 0.54 | 0.38 | |
| Subject 15 | Hugo | 0.14 | 0.12 | 0.08 | 0.08 | 0.31 | 0.25 | |
|  | Emma | 0.16 | 0.12 | 0.07 | 0.07 | 0.39 | 0.25 | |
|  | **Katja** | **0.15** | **0.11** | **0.06** | **0.07** | **0.39** | **0.24** | |
| Subject 16 | **Hugo** | **0.19** | **0.11** | **0.06** | **0.06** | **0.39** | **0.21** | |
|  | Emma | 0.22 | 0.15 | 0.05 | 0.05 | 0.49 | 0.31 | |
|  | Katja | 0.23 | 0.15 | 0.05 | 0.05 | 0.50 | 0.32 | |

**Figure S1.** Comparison of simulated and experimental $B_{1}^{+}$ magnitude/phase maps in subject 6 (sagittal, coronal and axial planes are shown, the black line is the experimental brain contour, segmented from the MPRAGE sequence): (a, e) simulation with another biomodel for this subject (Katja) and the standard brain conductivity ($\sigma_{ex-vivo}=0.46$ S/m) ; (b, f) simulation with the best biomodel for this subject (Emma) and the standard brain conductivity ($\sigma_{ex-vivo}=0.46$ S/m) ; (c, g) simulation with the best biomodel for this subject (Emma) and brain conductivity ($\sigma_{in-vivo}=0.70$ S/m) ; (d, h) experimental $B_{1}^{+}$ maps.


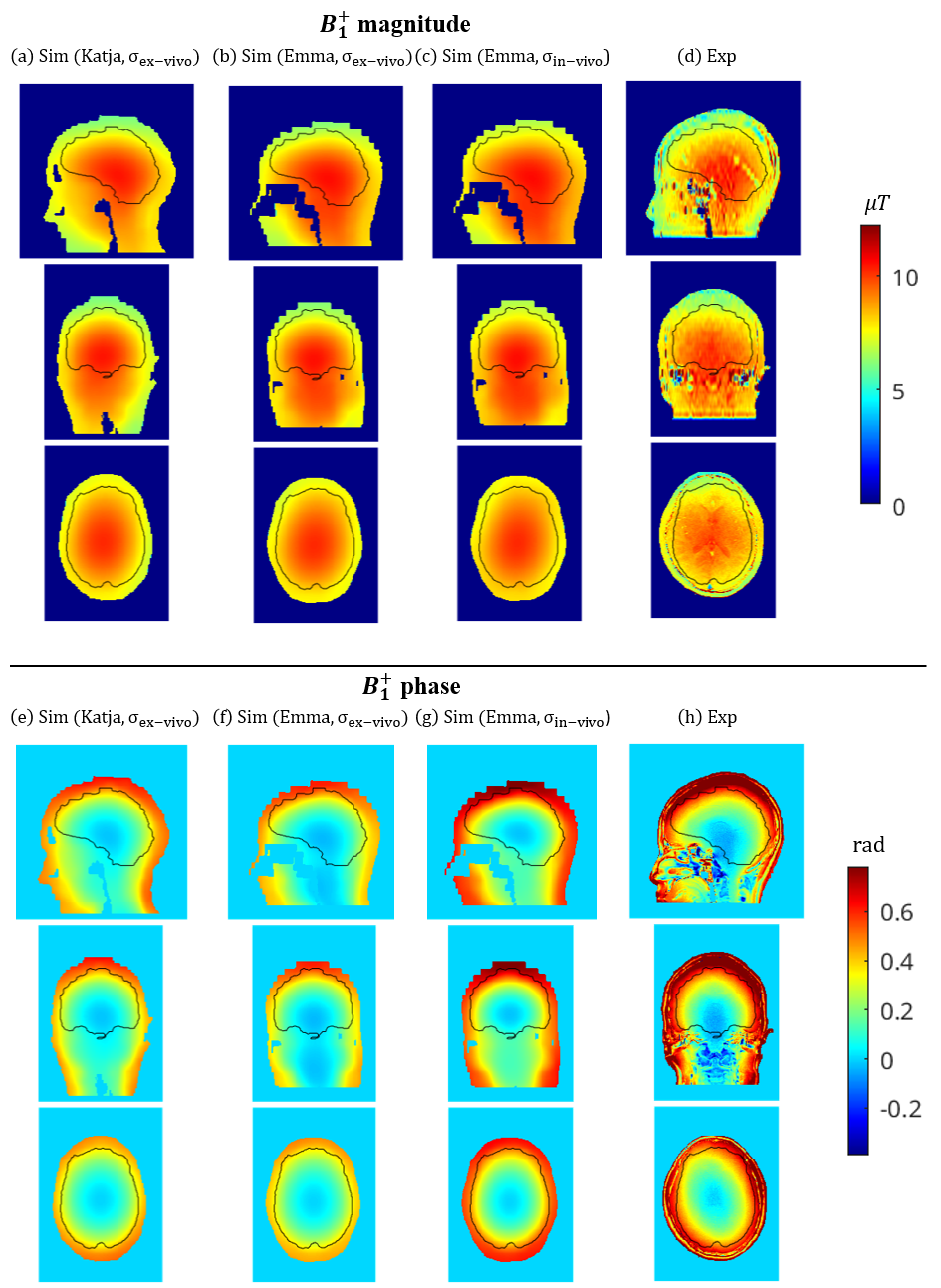


**Figure S2.** Comparison of SAR maps in subject 6, with different estimation methods (sagittal, coronal and axial planes are shown, the green line is the brain contour of the biomodel, in simulated maps, or the subject, in experimental maps): (a) simulated SAR using exact calculation and standard brain conductivity ($\sigma_{ex-vivo}=0.46$ S/m); (b) simulated SAR using $B_{1}^{+}$-derived estimation and standard brain conductivity; (c) experimental SAR using $B_{1}^{+}$-derived estimation and standard brain conductivity; (d, e, f) same as (a, b, c) but using brain conductivity $\sigma_{in-vivo}=0.70$ S/m. Here the biomodel used for simulation was Emma (best match for $B_{1}^{+}$ maps).


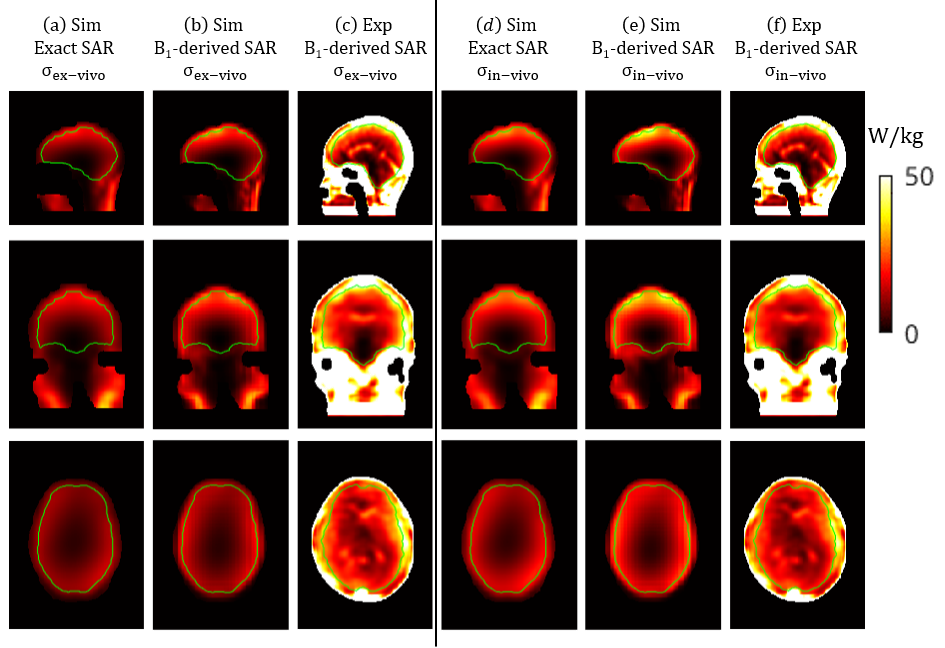


**Figure S3.** Comparison of simulated and experimental $B_{1}^{+}$ magnitude/phase maps in the subject with the highest NRMSE score for complex $B_{1}^{+}$ maps (subject 14). The NRMSE scores are 0.22 for $\sigma_{ex-vivo}$ complex map and 0.15 for $\sigma_{in-vivo}$ complex map. The mean NRMSE scores on all the subjects and their respective best fitting biomodel are 0.14 ± 0.03 for $\sigma_{ex-vivo}$ complex maps and 0.10 ± 0.02 for $\sigma_{in-vivo}$ complex maps. Sagittal, coronal and axial planes are shown, the black line is the experimental brain contour, segmented from the MPRAGE sequence: (a, e) simulation with another biomodel for this subject (Katja) and the standard brain conductivity ($\sigma_{ex-vivo}=0.46$ S/m) ; (b, f) simulation with the best biomodel for this subject (Hugo) and the standard brain conductivity ($\sigma_{ex-vivo}=0.46$ S/m) ; (c, g) simulation with the best biomodel for this subject (Hugo) and brain conductivity ($\sigma_{in-vivo}=0.70$ S/m) ; (d, h) experimental $B_{1}^{+}$ maps.


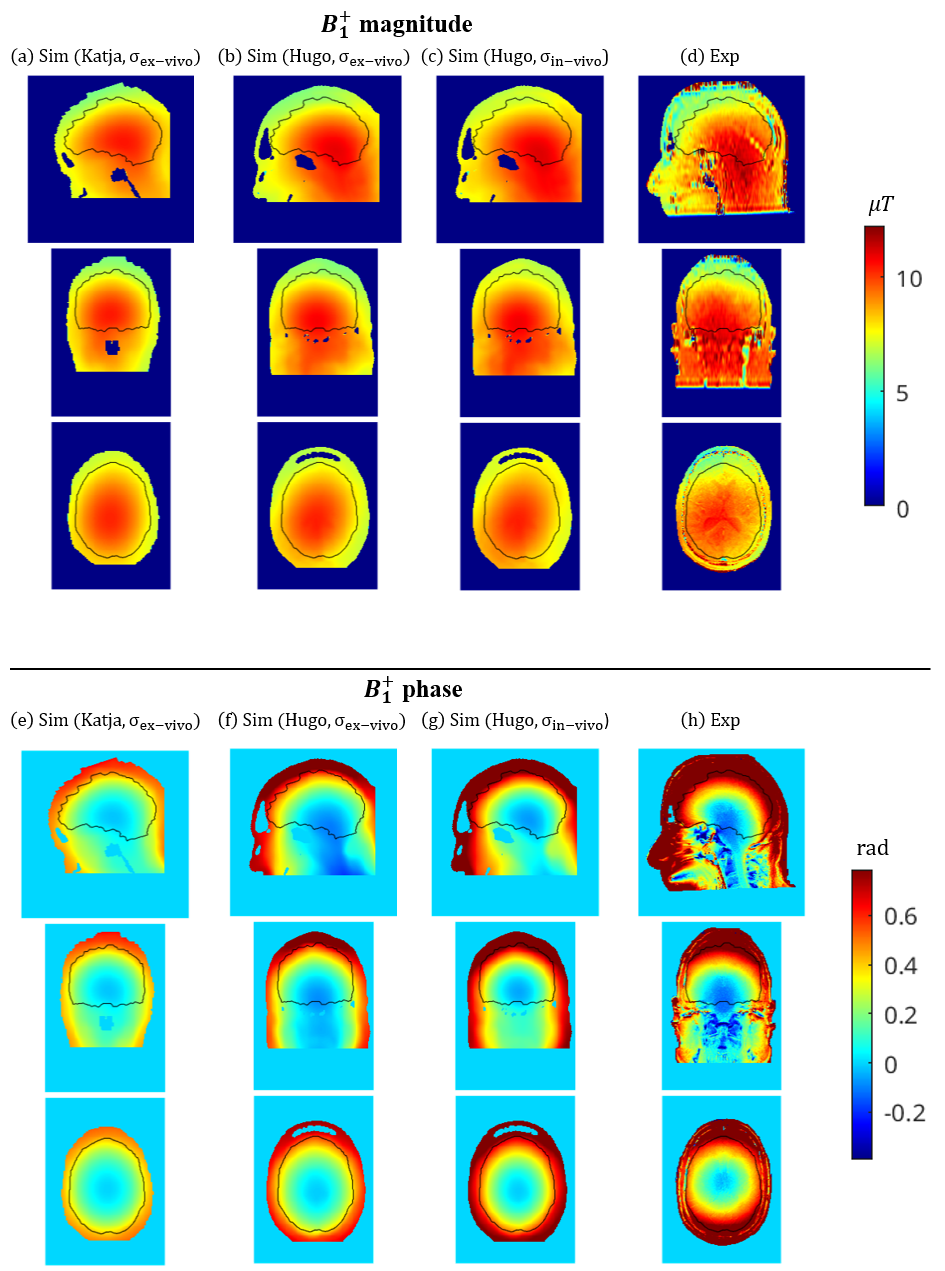


**Figure S4.** Comparison of SAR maps in the subject with the highest NRMSE score for complex $B_{1}^{+}$ maps (subject 14), with different estimation methods (sagittal, coronal and axial planes are shown, the green line is the brain contour of the biomodel, in simulated maps, or the subject, in experimental maps): (a) simulated SAR using exact calculation and standard brain conductivity ($\sigma_{ex-vivo}=0.46$ S/m); (b) simulated SAR using $B_{1}^{+}$-derived estimation and standard brain conductivity; (c) experimental SAR using $B_{1}^{+}$-derived estimation and standard brain conductivity; (d, e, f) same as (a, b, c) but using brain conductivity $\sigma_{in-vivo}=0.70$ S/m. Here the biomodel used for simulation was Hugo (best match for $B_{1}^{+}$ maps).


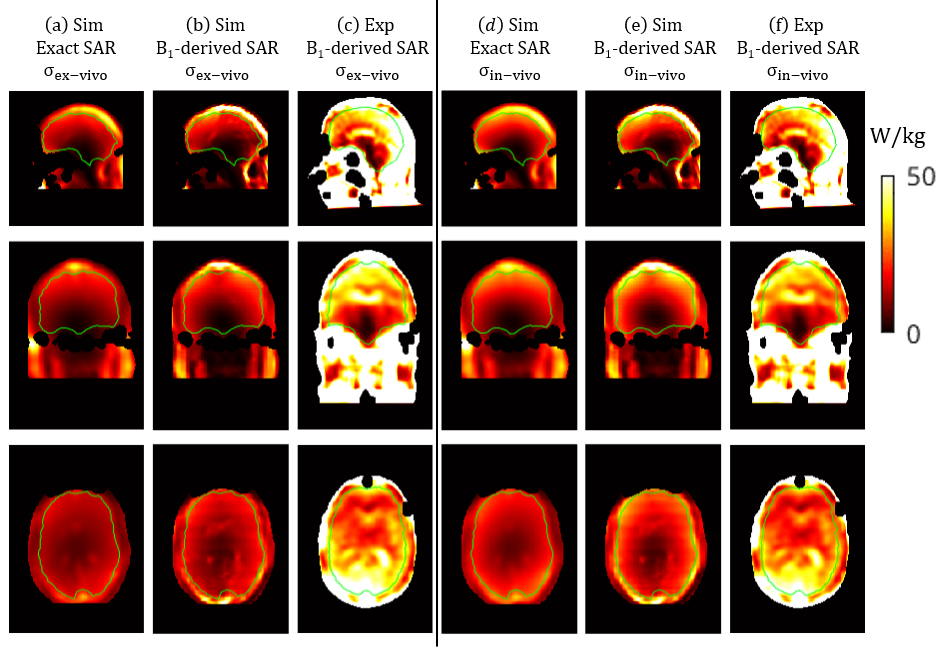

Supplement: Supplementary file 1 — Table S1: Main characteristics for all the subjects included in the study. Table S2: Normalized root‐mean‐squared error (NRMSE) between simulated B1+ maps and experimental B1+ maps for 16 healthy subjects, in the brain region, using different biomodels (Emma, Katja, and Hugo) and brain conductivity values (σexvivo or σin vivo). For each subject, the biomodel selected as “best biomodel” is in bold and was selected based on its NRMSE complex score. Figure S1: Comparison of simulated and experimental B1+ magnitude/phase maps in subject 6 (sagittal, coronal and axial planes are shown, the black line is the experimental brain contour, segmented from the MPRAGE sequence): (a, e) simulation with another biomodel for this subject (Katja) and the standard brain conductivity (σexvivo=0.46 S/m); (b, f) simulation with the best biomodel for this subject (Emma) and the standard brain conductivity (σexvivo=0.46 S/m); (c, g) simulation with the best biomodel for this subject (Emma) and brain conductivity (σin vivo=0.70 S/m); (d, h) experimental B1+ maps. Figure S2: Comparison of SAR maps in subject 6, with different estimation methods (sagittal, coronal and axial planes are shown, the green line is the brain contour of the biomodel, in simulated maps, or the subject, in experimental maps): (a) simulated SAR using exact calculation and standard brain conductivity (σexvivo=0.46 S/m); (b) simulated SAR using B1+‐derived estimation and standard brain conductivity; (c) experimental SAR using B1+‐derived estimation and standard brain conductivity; (d, e, f) same as (a, b, c) but using brain conductivity σin vivo=0.70 S/m. Here, the biomodel used for simulation was Emma (best match for B1+ maps). Figure S3: Comparison of simulated and experimental B1+ magnitude/phase maps in the subject with the highest NRMSE score for complex B1+ maps (subject 14). The NRMSE scores are 0.22 for σexvivo complex map and 0.15 for σin vivo complex map. The mean NRMSE scores on all the subjects and their res [file NBM-39-e70335-s001.docx]
